# Elucidating regulation of polyhydroxyalkanoate metabolism in *Ralstonia eutropha*: Identification of transcriptional regulators from phasin and depolymerase genes

**DOI:** 10.1016/j.jbc.2024.107523

**Published:** 2024-07-04

**Authors:** Lara Santolin, Rosalie Sandra Josianne Eichenroth, Paul Cornehl, Henrike Wortmann, Christian Forbrig, Anne Schulze, Inam Ul Haq, Sabine Brantl, Juri Rappsilber, Sebastian Lothar Riedel, Peter Neubauer, Matthias Gimpel

**Affiliations:** 1 Technische Universität Berlin, Chair of Bioprocess Engineering, Berlin, Germany; 2 Technische Universität Berlin, Chair of Bioanalytics, Berlin, Germany; 3 Matthias-Schleiden-Institut für Genetik, Bioinformatik und Molekulare Botanik, AG Bakteriengenetik, Friedrich-Schiller-Universität Jena, Jena, Germany; 4 Berliner Hochschule für Technik, Environmental and Bioprocess Engineering Laboratory, Berlin, Germany

**Keywords:** *Ralstonia eutropha*, polyhydroxyalkanoates, gene regulation, transcription factors, phasins, depolymerases

## Abstract

Despite the ever-growing research interest in polyhydroxyalkanoates (PHAs) as green plastic alternatives, our understanding of the regulatory mechanisms governing PHA synthesis, storage, and degradation in the model organism *Ralstonia eutropha* remains limited. Given its importance for central carbon metabolism, PHA homeostasis is probably controlled by a complex network of transcriptional regulators. Understanding this fine-tuning is the key for developing improved PHA production strains thereby boosting the application of PHAs. We conducted promoter pull-down assays with crude protein extracts from *R. eutropha* Re2058/pCB113, followed by liquid chromatography with tandem mass spectrometry, to identify putative transcriptional regulators involved in the expression control of PHA metabolism, specifically targeting phasin *phaP1* and depolymerase *phaZ3* and *phaZ5* genes. The impact on promoter activity was studied *in vivo* using β-galactosidase assays and the most promising candidates were heterologously produced in *Escherichia coli*, and their interaction with the promoters investigated *in vitro* by electrophoretic mobility shift assays. We could show that *R. eutropha* DNA-binding xenobiotic response element-family-like protein H16_B1672, specifically binds the *phaP1* promoter *in vitro* with a *K*_D_ of 175 nM and represses gene expression from this promoter *in vivo*. Protein H16_B1672 also showed interaction with both depolymerase promoters *in vivo* and *in vitro* suggesting a broader role in the regulation of PHA metabolism. Furthermore, *in vivo* assays revealed that the H-NS-like DNA-binding protein H16_B0227 and the peptidyl-prolyl cis-trans isomerase PpiB, strongly repress gene expression from P*phaP1* and P*phaZ3*, respectively. In summary, this study provides new insights into the regulation of PHA metabolism in *R. eutropha*, uncovering specific interactions of novel transcriptional regulators.

The stress-related storage of carbon and energy in the form of intracellular polyhydroxyalkanoate (PHA) granules is widely spread across prokaryotic species (1). PHA reservoirs also serve bacteria as electron sinks when facing oxygen limitation (2) and provide a shielding effect against various stresses such as UV radiation, oxidative and osmotic stress, and high/low temperatures (3). More than 70 bacterial and archaeal genera including *Cupriavidus, Pseudomonas, Azotobacter* species and many Haloarchaea accumulate the polymer to combat adverse environmental conditions (4). However, only a few organisms *e.g. Ralstonia eutropha* (also known as *Cupriavidus necator*), recombinant *Escherichia coli* or *Halomonas* sp. are used for industrial PHA production, with around 40% of annual PHA being produced in *R. eutropha* demonstrating its industrial relevance (5).

In *R. eutropha*, PHA accumulation is triggered by nutrient limitation or stress conditions under availability of excess carbon, and its synthesis involves three steps: (1) the condensation of two acetyl-CoAs by PhaA, (2) the reduction of acetoacetyl-CoA to 3-hydroxybutyryl-CoA by PhaB and, (3) the polymerization into polyhydroxybutyrate (PHB) by PHA synthase PhaC1. Extensive efforts have been made to modify *R. eutropha* to produce also other PHA polymers with enhanced thermoplastic properties (6); *R. eutropha* Re2058/pCB113 produces the PHA copolymer poly(hydroxybutyrate-*co*-hydroxyhexanoate) (P(HB-*co*-HHx)) from β-oxidation intermediates by expressing the (*R*)-specific enoyl-CoA hydratase (*phaJ*) and the heterologous synthase (*phaC2*), able to also incorporate 3-hydroxyhexanoate-CoA precursors into the PHA polymer (7). When synthetized, PHA aggregates in an amorphous state surrounded by several PHA granule-associated proteins forming the so called carbonosomes, typically 0.2 to 0.5 μm in diameter (2). Apart from PHA synthases (PhaCs, encoded in *phaC1* and *phaC2* genes), PHA depolymerases (PhaZs, encoded in *phaZ1-7* genes) and hydrolases (PhaYs, encoded in *phaY1* and *phaY2* genes) involved in PHA homeostasis, further PHA granule-associated proteins, which modulate the PHA content, granule size, and granule distribution in cells have been identified in *R. eutropha*: the phasins (PhaPs, encoded in *phaP1-8* genes), the regulator PhaR, and PhaM. Phasins are amphiphilic proteins which control the surface to volume ratio of the PHA granule. It was shown that, in a *phaP1* KO mutant, *R. eutropha* synthesizes less PHB in the form of only one very big PHB granule (8). Despite the great interest in engineering PHA metabolism, to date only one transcription factor (TF) regulating expression of PHA-related genes has been identified: protein PhaR binds the promoters of *phaP1* and *phaP3* and represses transcription of these genes. During PHA synthesis the repression is released as PhaR binds to nascent PHA granules. The transcription of *phaP1* and *phaP3* is inhibited again toward the end of PHA synthesis as PhaPs gradually cover the PHA granule that can no longer accommodate PhaR; excess PhaR also downregulates its own expression (4). Two further mechanisms related to the regulation of PHA metabolism have been described so far: one involving the physiological activator PhaM that forms an initiation complex with PhaC1 and attaches PHA granules to the nucleoid region being responsible for an equal distribution of PHA granules among daughter cells (9), and the second one related to the stringent response with reduced ppGpp alarmone levels leading to enhanced degradation of PHA suggesting that ppGpp acts as repressor of the PHA mobilization system (PhaZ1) (10).

Being involved in the central carbon metabolism, the intricate control of PHA metabolism can only be attributed to a complex network of transcriptional regulators. In the present study we performed pull-down assays with putative promoter regions of the phasin gene *phaP1*, and the depolymerase genes *phaZ3* and *phaZ5* and *R. eutropha* Re2058/pCB113 crude protein extracts followed by mass spectrometry (MS) analysis to identify potential transcriptional regulators of these genes. Interaction of the putative transcription factors with the promoters was confirmed by electrophoretic mobility shift assays (EMSAs) and regulation was elucidated by *in vivo* β-galactosidase reporter-gene assays.

## Results

### Identification of putative promoter sequences from *phaP* and *phaZ* genes

Phasins and PHA degrading enzymes are important for PHA storage and utilization. However, neither transcriptional regulators nor promoter sequences have been identified in *R. eutropha* for any of the phasin, depolymerase, or hydrolase encoding genes apart from *phaP1*. As a prerequisite for the search for transcriptional regulators, possible promoter sequences first had to be identified (see Figs. S1 and S2). For the search, we considered promoters that are dependent on either the housekeeping sigma factor or alternative sigma factors for general stress response and nitrogen metabolism. A σ^70^-like promoter sequence with almost perfect −35 and −10 boxes 147 bp upstream of the start codon, had already been described for *phaP1* (11). Additional putative σ^70^-like promoters could be identified for the *phaZ1*, *phaZ2*, and *phaZ3* genes encoding PHA depolymerases, however, with less perfect −35 and −10 boxes (see Fig. S3). For the phasin genes *phaP2*, *phaP4*, and *phaP5* as well as the depolymerase gene *phaZ6*, and hydrolase gene *phaY2* putative σ^S^ promoter sequences could be identified suggesting a stress-dependent regulation of some phasins and PHA degrading enzymes (Fig. S4). Except for the *phaP3* gene, between one and four possible σ^N^-binding sites could be detected for all phasin, depolymerase, and hydrolase genes (see Fig. S5). This is in line with the fact that nitrogen limitation is an important trigger for PHA synthesis. Surprisingly, for the *phaP3* gene neither σ^70^, σ^S^, or σ^N^ binding sites could be found. Based on the promoter predictions, 3′-biotin-tagged 100-mer oligonucleotides covering the proposed *phaP1, phaZ3*, and *phaZ5* promoters and their flanking regions were designed as prerequisite for the pull-down assays.

### Isolation of putative transcriptional regulators of phasin and depolymerase genes by promoter pull-down assays

**Figure 1:**
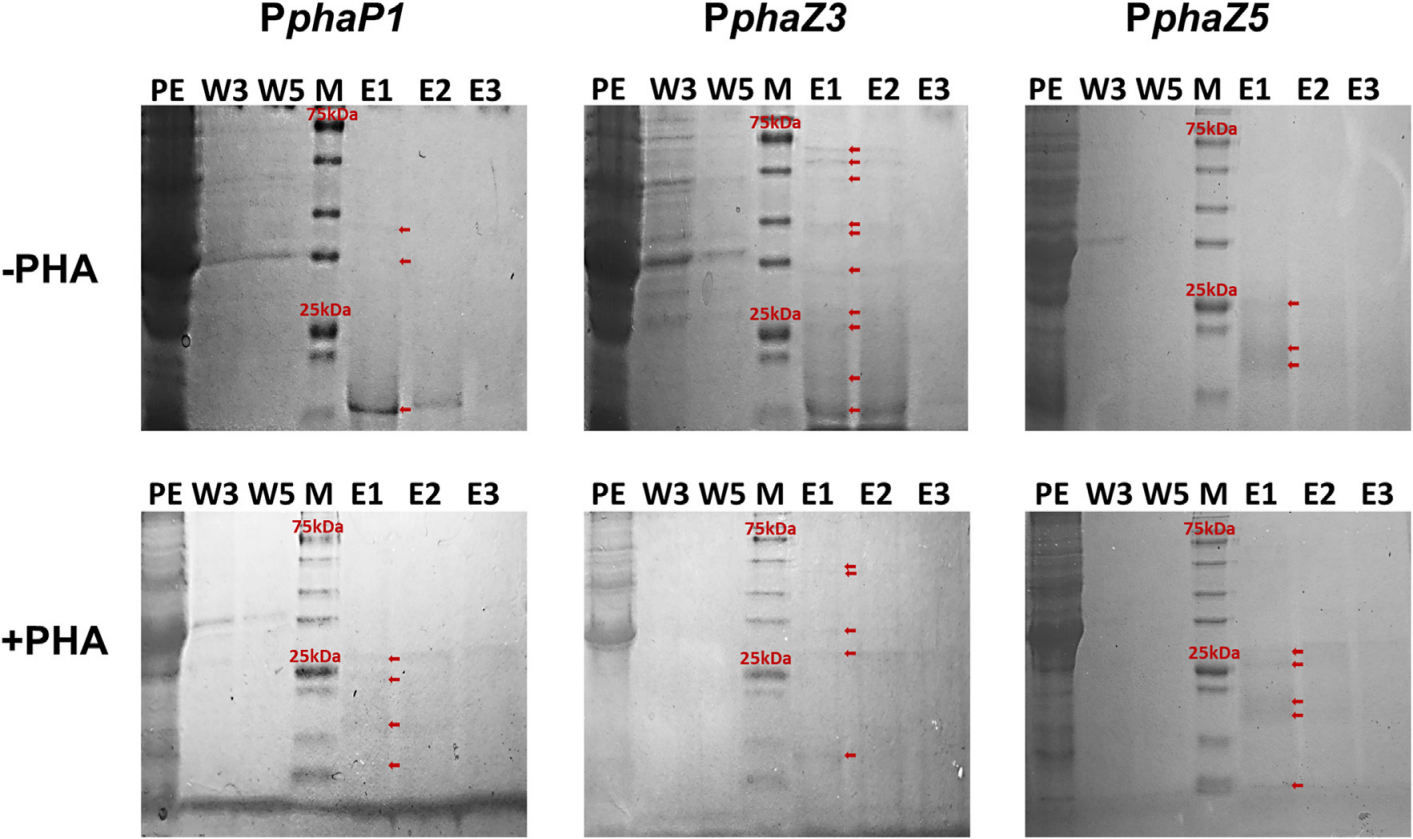
**Verification of protein enrichment during pull-down assays with promoter sequences of phasin gene *phaP1* and depolymerase genes *phaZ3* and *phaZ5*.** Protein extract (PE) was generated from 2 *Ralstonia eutropha Re2058/pCB113* cultivations (cultivated for 24 (-PHA) or 72 h (+PHA)) to map different PHA accumulation phases. Pull-down assays with P*phaP1*-streptavidin magnetic particles (SMP), P*phaZ3*-SMP, and P*phaZ5*-SMP were conducted as described in the section Experimental procedures. Subsequently, 5 μl PE and 15 μl of wash fractions (W) 3 and 5 and elution fractions (E) 1 to 3 were loaded on 12% polyacrylamide gels. Proteins were detected by Coomassie staining. *Red arrows mark* detectable eluted protein populations. In total, 5 μl pre-stained Roti Mark Tricolor (Carl Roth) was used as marker (M). PHA, polyhydroxyalkanoate.

In order to isolate proteins with high affinity to the putative *phaP1*, *phaZ3*, and *phaZ5* promoters, pull-down assays with *R. eutropha* Re2058/pCB113 growth phase (-PHA) and PHA accumulation phase (+PHA) crude protein extracts and biotin-tagged oligonucleotides coupled to magnetic beads were performed. Qualitative *in gel* analysis was conducted to verify the purification of proteins during pull-down assays (Fig. 1). While the protein content decreased in the washing fractions, distinct bands could be detected in the elution fractions indicating enrichment of proteins purified with each putative promoter region. The different band pattern observed in the elution fractions between noninduced basal PHA synthesis (P(HB-*co*-HHx) content of 18.5 wt% of cell dry weight with 30 mol% HHx; -PHA) and nitrogen-limitation-triggered PHA accumulation (P(HB-*co*-HHx) content of 60.3 wt% of cell dry weight with 26 mol% HHx; +PHA) as well as the different promoter fragments suggests specific interactions for each protein-promoter combination. Subsequently, the bound proteins were identified by liquid chromatography with tandem mass spectrometry (LC-MS/MS).

### Identification of purified proteins by LC-MS/MS

**Figure 2:**
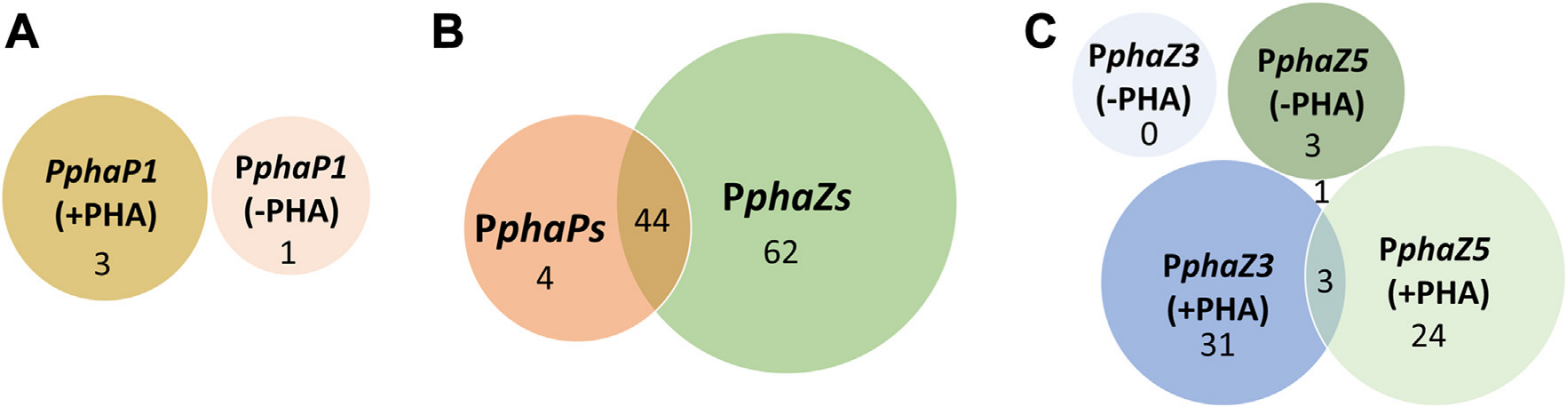
**Identification of putative transcription regulators of phasin and depolymerase genes.** Counts for identified promoter-bound proteins detected with an intensity above 1e^9^ (1 order of magnitude lower intensity compared to the internal control PhaR-P*phaP1* under PHA-basal conditions) are depicted. The area of the circles is proportional to the total hits for each condition, whereas the number inside the circles represent the number of proteins identified exclusively under each condition. *A*, identified proteins bound to the promoter of phasin gene *phaP1* under PHA-basal (-PHA) and PHA-accumulating conditions (+PHA). *B*, identified proteins bound to the promoter of phasin gene *phaP1* and depolymerase genes (*phaZ3* and *phaZ5*). *C*, identified proteins bound to the promoter of depolymerase genes *phaZ3* and *phaZ5* both in cell extracts under PHA-basal (-PHA) and PHA-accumulating (+PHA) conditions. PHA, polyhydroxyalkanoate.

A total of 1025 affinity-purified proteins from *R. eutropha* Re2058/pCB113 crude protein extracts could be identified by mass spectrometry (LC-MS/MS) (see Supplementary File raw MS data, all proteomics data have been deposited to the ProteomeXchange Consortium *via* the PRIDE: PXD050193): Proteins purified with P*phaP1* showed a total of 509 hits, with 56 being unique to this promoter. P*phaZ3* and P*phaZ5* showed 681 and 716 hits with 236 and 190 being unique, respectively. The transcriptional regulator PhaR, that is known to bind P*phaP1* under PHA inhibiting conditions, was detected with an intensity of 1.1e^10^ in protein fractions purified with P*phaP1* from crude protein extracts obtained during growth phase (PHA-). Interestingly, PhaR was also detected in all other protein fractions, except P*phaZ5* (+PHA), however with far less intensity (4e^7^ – 8e^8^). In order to filter out unspecific bindings, all identified proteins showing an intensity lower than 1e^9^ (10 times lower than the internal positive control PhaR) were deemed as nonspecifically bound, narrowing down total hits to 110. Clustering showed that only four of the identified proteins were exclusively purified with P*phaP1* whereas 62 were detected solely in P*phaZs* protein fractions (Fig. 2). Out of the four exclusively purified proteins with P*phaP1* only PhaR was bound under PHA inhibiting conditions (-PHA). Regarding P*phaZs* protein fractions, most were bound specifically to one promoter under one condition.

In order to further narrow down the transcriptional regulator candidates, ribosomal proteins, DNA-binding proteins involved in replication or DNA-repair, outer membrane proteins, and nucleases were excluded leaving a list of 47 candidates. Six transcriptional regulator candidates as well as the positive control PhaR (Table 1) were selected from the remaining list to be further evaluated in *in vitro* and *in vivo* experiments. Among the selected proteins were three members of known transcriptional regulator families, the H-NS-like DNA-binding proteins H16_B0227 and H16_B2256 that were identified in all protein fractions and the helix-turn-helix motif-containing xenobiotic response element (XRE)-family like protein H16_B1672 bound to P*phaP1* under PHA-accumulating conditions. Two proteins related to PHA metabolism were further selected. Firstly, the acetyl-CoA acetyltransferase PhaA, which was purified with higher intensities from PHA-accumulation protein fractions, and the *phaP1* encoded phasin PhaP1, which showed the highest intensity values of all identified proteins. Furthermore, the peptidyl-propyl cis-trans isomerase PpiB, a known protein chaperone that was detected in both P*phaP1* and P*phaZ5* protein fractions purified under PHA-accumulating conditions was included.

**Table 1:** Binding of putative transcriptional regulators to phasin and depolymerase promoters under PHA-basal (-PHA) or PHA-accumulating conditions (+PHA)

Putative transcriptional regulators		P*phaP1*		P*phaZ3*		P*phaZ5*	
		-PHA	+PHA	-PHA	+PHA	-PHA	+PHA
PhaR	Transcriptional regulator of *phaP1/P3*	**1.0e**^**+10**^	8.4e^+08^	2.2e^+08^	6.8e^+07^	4.1e^+07^	
H16_B0227	H-NS-like DNA-binding protein	**3.8e**^**+09**^	**5.2e**^**+09**^	8.7e^+08^	**2.1e**^**+09**^	2.3e^+08^	9.7e^+08^
H16_B2256	H-NS-like DNA-binding protein	**6.1e**^**+09**^	6.4e^+08^	**1.7e**^**+09**^	**2.6e**^**+09**^	2.1e^+08^	1.2e^+08^
H16_B1672	Helix-turn-helix XRE-family like protein		**1.1e**^**+09**^				
PhaA	Acetyl-CoA acetyltransferase	6.1e^+08^	**1.1e**^**+10**^	6.7^+08^	**1.7**^**+10**^	**1.5e**^**+09**^	**3.7e**^**+10**^
PhaP1	Phasin 1	**1.4e**^**+09**^	**8.7e**^**+10**^	9.9e^+08^	**1.5e**^**+09**^	**1.9e**^**+09**^	**1.4e**^**+11**^
PpiB	Peptidyl-propyl cis-trans isomerase	1.3e^+07^	**2.3e**^**+09**^	2.4e^+07^	6.9e^+06^	7.4e^+07^	**7.3e**^**+09**^

Abbreviation: LC-MS/MS, liquid chromatography with tandem mass spectrometry.

Label-free quantitation (LFQ) intensity of each hit determined by LC-MS/MS is displayed.

Intensities higher than 1.0∗e+09 (which corresponds to an intensity 1 order of magnitude lower than the internal positive control binding of PhaR to PphaP1) are highlighted in bold.

### Impact of putative transcriptional factors on expression from *R. eutropha* promoters *in vivo*

**Figure 3:**
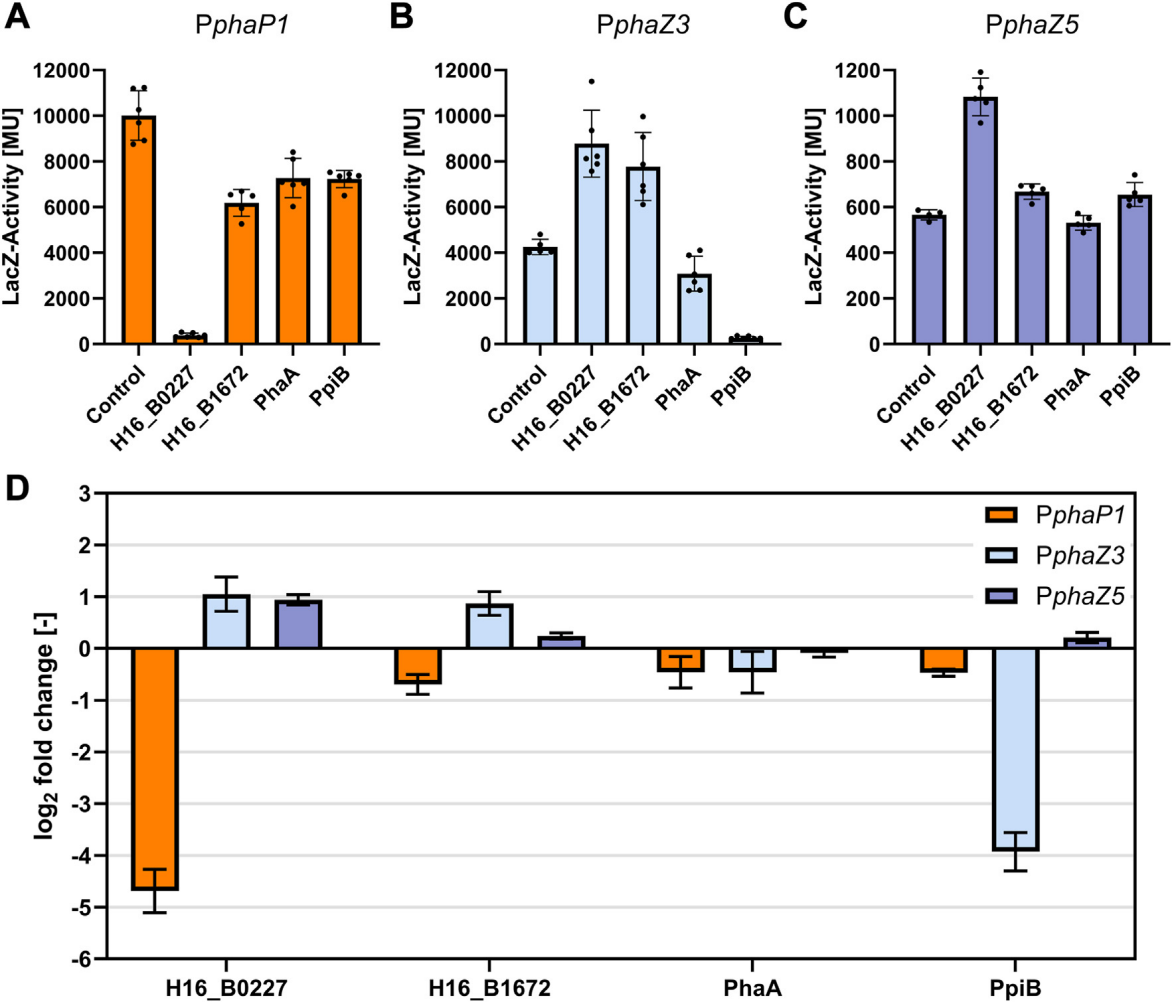
**Impact of putative transcriptional factors on expression from *Ralstonia eutropha* promoters P*phaP1*, P*phaZ3*, and P*phaZ5 in vivo*.***A*, impact on expression from P*phaP1* promoter. *B*, impact on expression from P*phaZ3* promoter. *C*, impact on expression from P*phaZ5* promoter.The measured β-galactosidase activity is displayed for each protein as well as the control with an empty plasmid for the putative TF. Scatter plots (*black dots*) are shown to report the reproducibility of 4 to 6 independent biological replicates within our data sets. *D*, the measured β-galactosidase activity is presented as a log_2_-fold change against a control with an empty plasmid for the putative TF. Error bars represent the standard deviation from 3 to 6 biological replicates. PHA, polyhydroxyalkanoate; TF, transcription factor.

The impact of potential transcriptional factors on gene expression from the *R. eutropha* phasin and depolymerase promoters was analyzed *in vivo* through a β-galactosidase reporter-gene assay (Fig. 3). Unfortunately, plasmids for overproduction of PhaP1 and H16_B2256 could not be constructed, hence leaving plasmids pGW5-B0227, pGW5-B1672, pGW5-PhaA, and pGW5-PpiB for overproduction of the putative TFs. Coexpression of the gene encoding the H-NS-like protein H16_B0227 resulted in a >25-fold repression of expression from the P*phaP1* promoter, while an about 2-fold activation was observed with both depolymerase promoters. Coexpression of the PpiB encoding gene resulted in a >15-fold repression of P*phaZ3*. Weaker effects were observed by coexpressing the gene encoding the helix-turn-helix domain-containing protein H16_B1672, that showed a negative effect on expression from P*phaP1* (>1.5 fold) and a weak activation of expression from P*phaZ3*. Co-expression of *phaA* did not show any significant effect on the expression from either tested promoter. Finally, coexpression of the genes encoding the four putative transcriptional regulators did not have any significant effect on the expression from the control promoter P*cggR*, further corroborating the specific interaction of the putative TFs with *R. eutropha* promoters (see Fig. S6).

### *In vitro* confirmation of promoter-binding by EMSAs

**Figure 4:**
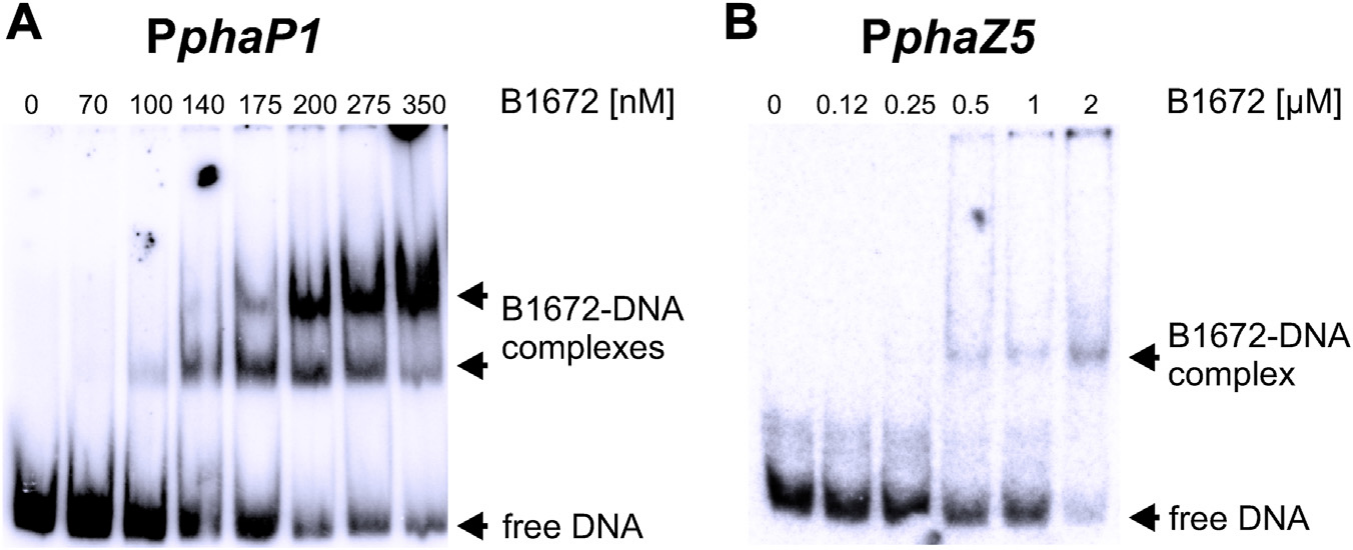
**Confirmation of*****in vitro*****binding.** Electrophoretic mobility shift assay for *in vitro* binding of putative transcription factor protein H16_B1672 to phasin promoter P*phaP1* (*A*) and depolymerase promoter P*phaZ5* (*B*).

The most promising transcriptional regulator candidates, H16_B0227, H16_B1672, and PpiB, were heterologously produced in *E.coli*, purified *via* a Strep-tag and their interaction with the phasin and depolymerase promoters was examined *in vitro* using EMSAs. Surprisingly, no binding to any promoter could be detected *in vitro* for H16_B0227 or PpiB (see Fig. S7) despite the strong effects observed *in vivo* (Fig. 3). In contrast, binding of protein H16_B1672 to phasin promoter P*phaP1* could be confirmed *in vitro* (Fig. 4*A*). With increasing protein concentrations, a decrease in free DNA was observed, while two bands indicating different protein-DNA complexes appeared. The equilibrium dissociation constant (*K*_D_) was estimated to be 175 nM. For the depolymerase promoter P*phaZ3* no binding was observed *in vitro* to protein H16_B1672 (see Fig. S8). Unexpectedly, H16_B1672 also bound to P*phaZ5*, although only at much higher protein concentrations with a *K*_D_ of about 1.5 μM (Fig. 4*B*). An additional EMSA with the heterologous *Bacillus subtilis* promoter P*cggR* confirmed the specificity of the observed interactions of H16_B1672 with *PphaP1* and *PphaZ5*, respectively (see Fig. S11).

## Discussion

Despite its scientific importance as a model organism for autotrophic growth or the presence of oxygen-tolerant [NiFe] hydrogenases and its industrial relevance as a PHA bioplastic producer (6), little is known about the regulation of transcription in *R. eutropha* or its DNA-binding regulatory proteins. In this study, we identified novel transcriptional regulators of PHA metabolism using pull-down assays followed by LC-MS/MS. To the best of our knowledge, this is the first time a promoter pull-down assay is applied to screen for regulators related to PHA metabolism, although it has broadly been used to identify other novel transcriptional factors (12, 13, 14, 15).

**Figure 5:**
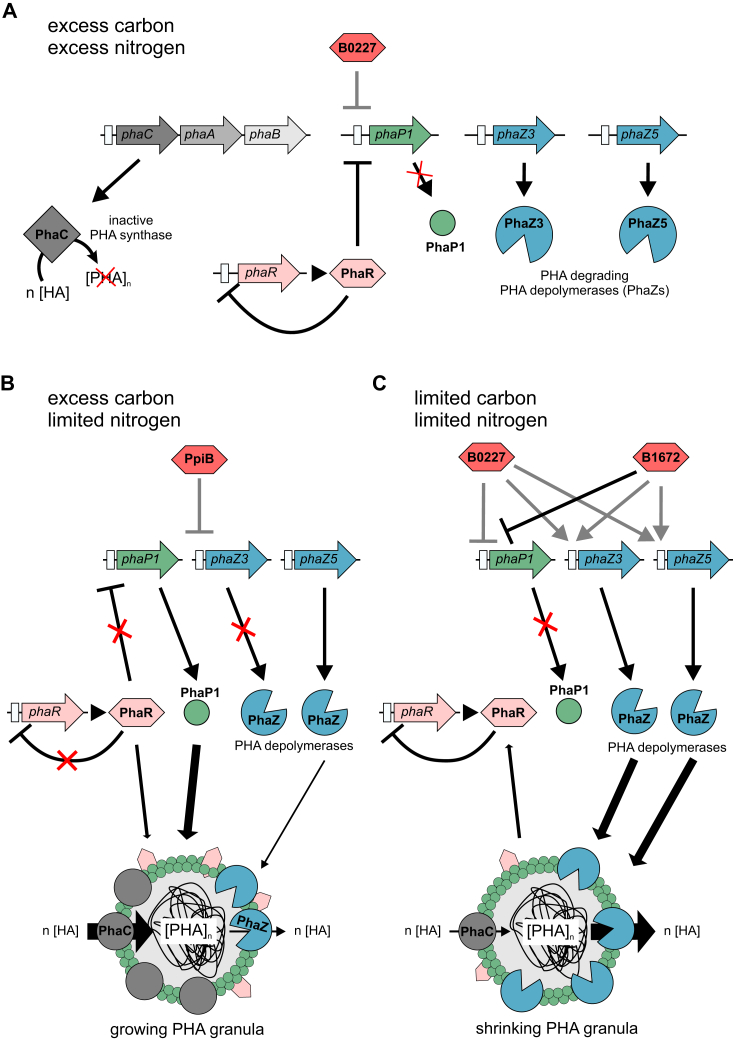
**Working model.***A*, in the presence of excess carbon and nitrogen the PHA synthase PhaC is inactive as PHA synthesis is not required. Expression of phasin *phaP1* is repressed by PhaR and B0227 while expression of the depolymerase encoding genes *phaZ3* and *phaZ5* remains at a basal level and is not further activated since B1672 is not present. *B*, when nitrogen becomes limiting the excess carbon is converted to PHA by the action of the PHA polymerase PhaC. The produced PHA is stored in PHA granules that are coated with phasins (PhaPs). Repression of the major phasin encoding *phaP1* gene is relieved as PhaR is relocated to the forming granules that are stabilized by the produced phasins. In addition, important proteins of the PHA metabolism *e.g.* the PHA synthase PhaC, and intracellular PHA depolymerases (PhaZs) are also associated with the PHA granules. To prevent rapid degradation of the forming PHA, expression of *phaZ3* encoding a PHA depolymerase is repressed by PpiB. *C*, at the end of the production phase carbon limitation results in the mobilization of the stored PHA. Under this condition composition of the granules changes. Expression of the PHA depolymerase encoding genes *phaZ3* and *phaZ5* is activated by B0227 and B1672, while, *phaP1* expression is repressed by B0227 and B1672. PhaR is only slowly released from the PHA granules and not yet contributes to regulation of *phaP1*. *Arrows*: activation; *blocked arrows*: repression; *Black arrow*: effects observed with both β-galactosidase assay and EMSA; *Gray arrow*: effects observed either with β-galactosidase assays or EMSA, *Green circles*: phasins; *Gray circles*: PHA polymerase PhaC; *Blue circles*: PHA depolymerase; *Hexagons*: transcriptional regulators. EMSA, electrophoretic mobility shift assay; PHA, polyhydroxyalkanoate.

Our findings suggest that the helix-turn-helix domain-containing protein H16_B1672 is a novel TF in *R. eutropha* that contributes to the regulation of the phasin PhaP1, most probably towards the end of the PHA accumulation phase (Fig. 5). Supporting this, we could show that H16_B1672, that was exclusively pulled down with the *phaP1* promoter under PHA-accumulating conditions, specifically interacts with P*phaP1 in vitro* with an approximate *K*_D_ of 175 nM, whereas complementary *in vivo* reporter gene assays indicate a repressing function of the protein on the phasin promoter as shown in our model, presumably B1672 is involved in repression of *phaP1* expression at the end of the PHA accumulation phase (Fig. 5*C*). The function of H16_B1672 in *R. eutropha* has not been described so far, nevertheless sequence analysis indicates that it belongs to the XRE transcriptional regulator family. Another XRE-family protein, *E. coli* SutR, is a known transcription factor with a regulatory role in sulfur utilization (16). Unexpectedly, EMSAs showed that the protein also binds the depolymerase promoter P*phaZ5 in vitro* although no significant effect on expression from this promoter was observed *in vivo*. Although the *in vitro* binding affinity to P*phaZ5* was much lower than to P*phaP1*, the complex formation showed to be specific as it was not disturbed when heterologous DNA was added, which is usually tested to verify specific complex formation (17). In contrast, despite a weak activation effect of H16_B1672 on transcription from the depolymerase promoter P*phaZ3* was noted *in vivo*, no binding could be observed *in vitro*. However*, in vitro* binding and *in vivo* effects of H16_B1672 on the depolymerase promoters suggest a broader role of the protein in the regulation of PHA metabolism (Fig. 5*C*) that requires further investigation.

*In vivo* β-galactosidase reporter gene assays in *E. coli* have been used to study the impact of transcriptional factors on expression from *R. eutropha* promoters in the past (18). Coexpression of the gene encoding the second TF candidate, protein H16_B0227, which is an H-NS-like DNA-binding protein, strongly repressed gene expression from the phasin promoter P*phaP1* (>25 fold) whereas it showed a weak induction of expression from depolymerase promoters P*phaZ3* and P*phaZ5*. The nearly uncharacterized *R. eutropha* H16_B0227 was highly abundant in all protein fractions identified by LC-MS/MS and therefore, seems to be a promising candidate for transcriptional regulation of phasins and depolymerases. DNA-binding capabilities of this protein were already characterized as a histone-like binding protein that binds AT-rich dsDNA and, with lower affinity, also RNA and ssDNA (19). Unfortunately, binding of H16_B0277 to *R. eutropha* promoters could not be verified *in vitro*. As protein-DNA interactions are sensitive to osmolarity, pH, temperature, and some may need cofactors to form complexes (20), improved EMSA conditions might lead to a successful complex formation. Since the tertiary structure of a protein is essential for its specific DNA binding (21), the failing of the EMSA could be due to misfolding of the heterologously produced H16_B0227, its inactivation during purification or storage, or the missing of an essential cofactor. H-NS-like proteins are known to form heteromeric and homomeric complexes which can affect binding to DNA (22). The presence of B0227 after both 24 and 72 h of cultivation suggests a broader role for the protein in regulation of *phaP1* expression. It is conceivable that B0227 together with PhaR regulates the expression of *phaP1* during the growth phase, when no PHA is produced, whereas it suppresses the expression of the *phaP1* gene together with B1672 after completion of PHA production when no further phasins are required (Fig. 5). An involvement of PhaR in the suppression of *phaP1* expression after 72 h cannot be completely excluded. Our pull-down experiments showed that PhaR also bound to P*phaP1* after 72 h (Table 1), although at a significantly lower concentration. A possible explanation could be heterogeneity within the population after 72 h. In a part of the population the PHA synthesis is not yet completed and therefore further phasins are needed for the growth of the granules. In this part of the population, PhaR is associated with the growing granules and is not available for the repression of *phaP1* while B1672 has not yet been formed (Fig. 5*B*). In contrast, another part of the population has already finished PHA production so that no further phasins are required. Here, the expression of *phaP1* is blocked by B1672, which is now present together with B0227. In this time window, B1672 might take over the function of PhaR while PhaR is only slowly released from the shrinking granules and can therefore only gradually take over the repression of *phaP1* again (Fig. 5*C*). Similarly, upon completion of PHA production, B0227 and B1672 can upregulate the expression of PHA depolymerases in a cooperative manner (Fig. 5*C*) which has been described for numerous other systems (23).

The metabolic enzyme peptidyl-propyl cis-trans isomerase PpiB, that was pulled with high abundances in protein fractions during PHA production, imparted a strong repression of expression from the depolymerase promoter P*phaZ3* (>15 fold) *in vivo*. Furthermore, coexpression of the gene encoding PpiB did not influence *lacZ* expression under the control of P*cggR*, indicating a specific effect of PpiB on P*phaZ3* that is not related to PpiB chaperone activity. PPIs such as PpiB are well-known moonlighting proteins that, in eukaryotes, are involved in RNA splicing in addition to the isomerization of peptide bonds (24), and participate in virulence in pathogenic prokaryotes (25). Protein PpiB in *R. eutropha* could moonlight as a repressor of the depolymerase *phaZ3* inhibiting PHA mobilization during PHA accumulation (Fig. 5*B*). Supporting this hypothesis, it has already been suggested that some PPIs could modulate gene transcription as some possess domains typical of TFs (26); however, direct DNA binding of PpiBs has not yet been reported. Nevertheless, *in silico* structure prediction of PpiB (data not shown) indicated that positively charged α-helices are exposed on the surface of the protein, which could allow DNA binding through electrostatic interactions with the negatively charged phosphate backbone.

Recently, a participation of PII proteins in regulation of PHA metabolism was reported in *Synechoscystis* sp. (27, 28) and *Herbaspirillum seropedicae* (29). These proteins participate in a signal transduction cascade that modulates the activity of transcriptional regulators in response to the availability of nitrogen (29). Similar interactions of the transcriptional regulators found in our study cannot be ruled out, but where not yet investigated.

Using the promoter region of the phasin gene *phaP1* for the pull-down assay, the protein PhaR could be purified from *R. eutropha* Re2058/pCB113 growth phase protein crude extracts with high relative abundance, further corroborating that PhaR binds the promoter P*phaP1* under PHA inhibiting conditions (11, 30). PhaR is the only transcriptional regulator known to date that controls expression of PHA genes in *R. eutropha* (4).

As a prerequisite for promoter pull-down assays, we predicted putative promoters upstream of phasin and depolymerase encoding ORFs. In addition to the well-known σ^70^-like promoter of the *phaP1* gene (11), several σ^70^, σ^S^, and σ^N^-like promoters could be predicted in the upstream region of the phasin and PHA depolymerase and hydrolase encoding genes which is not surprising as PHA accumulation is majorly triggered under nutrient limitation. In most cases more than one putative sigma factor binding site could be predicted suggesting alternative transcriptional start sites that might indicate regulated expression under different conditions. Compared to the σ^70^-like promoter of the *phaP1* gene, all predicted putative promoters showed less sequence identity with the consensus promoter, thus suggesting the need for additional transcription factors for activation of transcription which is in line with the huge number of putative DNA binding proteins enriched in our pull-down assays. The different set of putative interacting proteins and the difference in the upstream regions of phasin, depolymerase, and hydrolase encoding genes despite the functional redundancy of the encoded proteins indicates a conditional regulation of PHA storage and utilization. To achieve such a regulation a specific set of transcriptional regulators is required which inspired the current work.

For pull-down assays and *in vivo* and *in vitro* experiments, a DNA region upstream of each gene where likely most TF binding sites would be enclosed was selected. In this study, the DNA region 100 nt upstream the proposed transcription start site of each gene was used. However, we are aware that a primer extension assay would be necessary to accurately determine the transcription start site of each gene (31). Also, it is known that some TFs bind downstream the transcription start site or even further upstream (11, 32) and therefore, the use of longer DNA sequences might lead to the identification of further TFs.

## Conclusion

Even though all of the transcription regulator candidates have been enriched in the pull-down assays and most of the tested candidates showed a specific impact on transcription using *in vivo* reporter gene assays, only for H16_B1672 a specific DNA-protein interaction could be verified *in vitro*. The difficulty in the search for transcription factors seems to be less associated to their detection than to their functional characterization. A major problem seems to be the heterologous production of these proteins for the *in vitro* experiments required. Nevertheless, our results are a first step toward a better understanding of the regulatory network behind PHA metabolism in *R. eutropha* that will help to improve PHA production for industrial application. However, future work is required to completely understand the regulation of PHA synthesis, storage, and degradation as there are many more phasin and PHA depolymerase and hydrolase encoding genes in *R. eutropha* whose transcriptional regulation has not yet been investigated.

## Experimental procedures

### Bacterial strains and media

*R. eutropha* Re2058/pCB113, which is derived from *R. eutropha* H16 (American Type Culture Collection 17699) and produces P(HB-*co*-HHx) when grown on oleaginous substrates (7), was used for the production of protein crude extracts. *E. coli* DH5α (33) was used for plasmid propagation and as a host for reporter gene assays, *E. coli* BL21 Gold (Agilent) was used for the heterologous production of putative TFs. All used strains are listed in Table S1.

Transformations were performed on solid and liquid TY medium (16 g L^−1^ tryptone, 10 g L^−1^ yeast extract, 5 g L^−1^ NaCl, for solid medium 2% (w/v) agar-agar). Plasmid propagations were conducted in TFB medium (12 g L^−1^ tryptone, 24 g L^−1^ yeast extract, 4 ml L^−1^ glycerol, 0.23 g L^−1^ KH_2_PO_4_, 1.25 g L^−1^ K_2_HPO). EnPresso B medium (EnPresso GmbH) was used for the heterologous production of the putative TFs according to the manufacturer’s instructions. If required, all media were supplemented with 125 μg mL^−1^ ampicillin and/or 100 μg mL^−1^ spectinomycin.

### Preparation of protein crude extracts for pull-down assays

In order to identify transcriptional regulators of phasin and PHA depolymerase genes expressed during *R. eutropha* growth phase and PHA-accumulation phase, protein crude extracts were obtained from *R. eutropha* Re2058/pCB113. Cultivations were conducted in 2 L Ultra Yield Flasks (Thomson Instrument Company) containing 0.5 L mineral salts medium complemented with 60 g L^−1^ canola oil (Edeka Zentrale AG & Co KG,) and 4.48 g L^−1^ urea as described recently (34). The flasks were inoculated with 5 ml of a tryptic soy broth preculture at *A*_600_ = 5, covered by an AirOtop membrane (Thomson Instrument Company) and incubated at 200 rpm and 30 °C for 24 h (growth phase) or 72 h (PHA-accumulation phase) (25 mm amplitude, Multitron Standard, Infors AG). Cultures were harvested by centrifugation at 6500*g* for 15 min at 4 °C. Content and composition of PHA were determined from lyophilized cells (Gamma 1–20, Martin Christ Gefriertrocknungsanlagen GmbH) using a methanolysis protocol and gas chromatography as described previously (35). Crude protein extracts were obtained as followed: cell pellets were washed three times by resuspending in 30 ml ice-cold deionized water and 10 ml ice-cold *n*-hexane and subsequently centrifuging at 6500*g* for 15 min at 4 °C to remove residual oil from the cultivation. Subsequently, 15 mg of cells were resuspended in 25 ml extraction buffer (20 mM EDTA-Na_2_, 30 mM NaCl, 10 mM β-mercaptoethanol, 37.6 mM K_2_HPO_4_, 2.4 mM KH_2_PO_4_, and 10 mM of protease inhibitor PMSF). A French press (SLM AMINCO) with a pressure of 1000 PSIG was used for lysis for four passages. Cell extracts were centrifuged for 20 min at 4 °C and 6500*g* and the supernatant (protein extract) was stored at −20 °C.

### Identification of putative promoter sequences from *phaP* and *phaZ* genes and promoter-encoding oligonucleotide synthesis

Sequences of phasin (*phaP1-7*) and PHA depolymerase encoding genes (*phaZ1-8*) were obtained from the NCBI data bank (Accession number: AM260479, AM260480; see Table S2). Putative promoters in the upstream region of the ORF were predicted by manually searching for putative housekeeping promoters with a −35/-10 consensus motif [TTGACA(N)_17±1_TATAAT], putative nitrogen dependent promoters with the −24/-12 consensus sequence [YGGMYR(N)_4±1_YYGCW], or putative stress dependent promoters with a −35/-10 consensus sequence [CCGGCG(N)_17±1_CTATACT]. Sequences matching the respective consensus sequence with up to four mismatches were considered as putative promoters. 3′-biotin tagged as well as free single-stranded oligonucleotides with a length of 100 nt, covering the selected putative promoters of *phaP1*, *phaZ3*, and *phaZ5* as well as flanking regions (see Table S4), were chemically synthesized (Merck KGaA).

### Pull-down assays

In order to isolate putative transcriptional regulators of *phaP* and *phaZ* genes, streptavidin-coated magnetic beads (Streptavidin MagneSphere Paramagnetic Particles [SMP], Promega) were loaded with biotin-tagged oligonucleotides covering putative promoter regions and incubated with crude protein extracts obtained either from the *R. eutropha* growth phase or the PHA accumulation phase. To obtain double-stranded DNA oligos, single-stranded biotinylated oligonucleotides were mixed with their complementary nonbiotinylated strand in a 1:2 ratio, incubated at 95 °C for 5 min to dissolve secondary structures and slowly cooled to 30 °C to form dsDNA. Next, the dsDNA oligos were bound to streptavidin-coated magnetic beads; 1 mg of SMP (binding capacity of 0.75–1.25 nmol) were equilibrated and washed three times with 1 ml TEN100 buffer (10 mM Tris–HCl pH 7.5, 1 mM EDTA-Na_2_, and 100 mM NaCl) and finally resuspended in 400 μl TEN100. Subsequently, the prepared beads were incubated together with 3 nmol biotinylated annealed oligonucleotides for 25 min at 25 °C in an overhead shaker (Rotator Drive STR4, Stuart Scientific, Cole-Parmer). Oligonucleotide-coupled SMP was washed twice with 500 μl TEN1000 (10 mM Tris–HCl pH 7.5, 1 mM EDTA-Na_2_ and 1 M NaCl), followed by two washing steps with 500 μl 0.5× TBE buffer (89 mM Tris, 89 mM borate and 20 mM EDTA-Na_2_) and finally resuspended in 500 μl 0.5× TBE. Verification of hybridization of oligonucleotides and binding to SMP was performed by *in gel* analysis (data not shown). For pull-down assays, 50 μl of each oligonucleotide-coupled SMP were mixed with 1 ml of each protein extract complemented with 3.8 μg of salmon sperm DNA (Invitrogen, Thermo Fisher Scientific) and 0.5× TBE buffer and incubated in the overhead shaker for 1 h at 8 °C. SMPs were washed five times with 500 μl TGE buffer (44.5 mM Tris, 44.5 mM borate, 1 mM EDTA-Na_2_, 1.8 μg L^−1^ salmon sperm DNA, 20% (v/v) glycerin, 20 mM EDTA-Na_2_, 30 mM NaCl, 10 mM β-mercaptoethanol, 37.6 mM K_2_HPO_4_, and 2.4 mM KH_2_PO_4_). Elution was conducted by heating at 95 °C with 100 μl 25 mM desthiobiotin for 5 min. Eluted proteins were evaluated by SDS-PAGE and subsequent Coomassie staining.

### Identification of purified proteins by mass spectrometry

For MS-based identification of putative transcription factors, elution fractions of SMP-P*phaP*-/P*phaZ*-bound proteins were separated under denaturing conditions in a 10% precast polyacrylamide gel (Bio-Rad). The protein lanes were excised and cut into 1 mm^3^ cubes. The gel pieces were washed three times with 50% (v/v) acetonitrile (ACN) and 50 mM ammonium bicarbonate (ABC). Reduction of disulfide bonds was achieved by incubation for 30 min at 37 °C with 10 mM DTT in 50 mM ABC buffer. The free sulfhydryl groups within the sample were then alkylated by 20 min incubation with 55 mM iodoacetamide in 50 mM ABC buffer at 22 °C in the dark. Protein digestion was performed overnight at 37 °C (5 mg L^-1^ trypsin, 50 mM ABC buffer, and 5% (v/v) ACN) and later stopped by acidification to pH of 2 − 3 by the addition of 10% (v/v) TFA. Gel pieces were incubated with shaking for 15 min to extract peptides before loading on a self-prepared and conditioned C18-StageTip (36). The StageTip was washed two times with 50 μl 0.1% (v/v) TFA before eluting peptides with 0.1% (v/v) TFA and 80% (v/v) ACN. The solvent was removed from the eluate in a rotary evaporator and peptides were then dissolved in 0.1% (v/v) TFA and 1.6% (v/v) ACN for MS analysis on a Q Exactive HF hybrid quadrupole-Orbitrap mass spectrometer coupled on-line to an Ultimate 3000 RSLCnano Systems (Thermo Fisher Scientific). Peptides were separated on a 50 cm C18-EASY-Spray column (75 μm internal diameter, particle size: 2 μm) at 45 °C with a flow-rate of 0.3 μl min^−1^. The mobile phase consisted of buffer A (0.1% (v/v) formic acid) and buffer B (80% (v/v) ACN, 0.1% formic acid. The gradient started with 2% B and was increased to 5% B in 1 min, to 7.5% B in 2 min to 32.5% B in 32 min, to 38% B in 5 min to 45% B in 2.5 min to 52.5% B in 1.5 min, and then to 90% B in 1 min. MS data were acquired in data-dependent mode. MS1 spectra were recorded at 120,000 resolution (scan range 350– 1600 m/z). In each acquisition cycle, the ten most intense peaks with a charge between 2 and 6 were individually isolated with a 1.6 m/z window. The isolated ions were fragmented using higher-energy collisional dissociation with stepped collision energy (27%, 29%, and 31%). The maximum injection time for MS1 scans was set to 50 ms and the automatic gain control to a target of 3e^6^ ions. The MS2 spectra were recorded at a resolution of 15,000 with a maximum injection time of 80 ms and an automatic gain control target set to 1e^5^ ions. Dynamic exclusion was enabled for 30 s. Protein identification and label-free quantitation was conducted using MaxQuant (https://www.maxquant.org/) version 1.6.12.0 (37) with standard settings and the *R. eutropha* reference proteome (UP000008210, (38). Proteomic data are deposited in ProteomeXchange with accession PXD050193 (Username: reviewer_pxd050193@ebi.ac.uk with password: q4r4he0U).

### Construction of plasmids

Plasmid pGW5 (Gimpel, unpublished; all used plasmids are listed in Table S3), allowing inducible production of N terminally Strep-tagged proteins under control of the IPTG inducible P*lac* promoter was used as basis for construction of overexpression plasmids for heterologous production of putative transcriptional regulators. Candidate genes (*H16_B0227, H16_B1672, H16_B2256, phaA, phaP1*, and *ppiB*) were PCR amplified using primer pairs MG0264/MG0265, MG0290/MG0291, MG0288/MG0289, MG0258/MG0259, MG0292/MG0293, and MG0298/MG0299, respectively (all used oligonucleotides are listed in Table S4) using chromosomal DNA from *R. eutropha* H16 as the template. The PCR products were digested with BamHI and HindIII and subsequently ligated into pGW5 cut with the same enzymes yielding plasmids pGW5-B0227, pGW5-B1672, pGW5-B2256, pGW5-PhaA, pGW5-PhaP1, and pGW5-PpiB, respectively. A schematic map of all plasmids can be found in Fig. S10.

For the construction of reporter-gene fusions, 250 bp fragments covering the putative depolymerase gene promoters, P*phaZ3* and P*phaZ5*, were PCR amplified using primer pairs MG0278/MG0279 and MG0370/MG0372, respectively and chromosomal DNA from *R. eutropha* H16 as template. For construction of a P*phaP1*-*lacZ* fusion the phasin promoter fragment was amplified as follows. First, chromosomal DNA from *R. eutropha* H16 was used as template in a PCR with primer pair MG0310/MG0341. The fragment was purified and applied as template for a second PCR with primer pair MG0310/MG0342 yielding the P*phaP1* promoter region. As a control, the heterologous *B. subtilis* promoter P*cggR* was amplified using the primers MG0343/MG0346 and plasmid pMG7 (39) as template. The fragments were digested with EcoRI and BamHI and inserted into the medium-copy plasmid pGK-lacZ (Gimpel, unpublished) in front of the promoterless *lacZ* gene, resulting in plasmids pGK-P*phaZ3*-lacZ, pGK-P*phaZ5*-lacZ, pGK-P*phaP1*-lacZ, and pGK-P*cggR*-lacZ. Correctness of all inserts was verified by sequencing (LGC genomics). A schematic map of all plasmids can be found in Fig. S11.

### Protein purification

Purification of the Strep-tagged proteins using high-capacity Strep-Tactin Superflow (IBA) was performed according to the manufacturer’s instructions. Briefly, cell pellets were resuspended in Buffer W (100 mM Tris/HCl pH 8, 150 mM NaCl, 1 mM EDTA) (5 ml per g wet cells) and 1 mM protease inhibitor PMSF. Cells were disrupted by sonication (3 min, 30 s on/off, sonotrode with 7 mm diameter, 40% amplitude) (UP200S, Hielscher GmbH). Crude extracts were centrifuged for 20 min at 13,000*g* at 4 °C, and the soluble fraction was loaded onto a gravity flow Strep-Tactin column. The column was washed with five bed volumes of Buffer W, and the proteins were eluted six times with half column volume Buffer E (buffer W containing 2.5 mM D-desthiobiotin). The wash and elution fractions were collected and stored at −20 °C. SDS-PAGE and subsequent quantification of the bands with ImageJ (https://imagej.net/ij/) were used for determination of protein concentrations. A solution of bovine serum albumin served as standard. All gels were visualized by staining with colloidal Coomassie blue G250.

### β-galactosidase reporter gene assay

For reporter-gene-assays, *E. coli* DH5α was sequentially transformed with a pGK-LacZ derivative for *lacZ* gene expression under the control of the promoter to be tested and a pGW5 derivative encoding either of the putative TFs. Single colonies from a fresh transformation plate were used to inoculate precultures in 3 ml TY with 1 mM MgSO_4_ in 24-deep-well-plates with square shape wells (Duetz-MTPS, Adolf Kühner AG) and shaken overnight at 250 rpm and 37 °C (25 mm amplitude, Multitron Standard, Infors AG). For the main culture, each well of an analogously aliquoted deep-well-plates was inoculated to an *A*_600_ = 0.2 and cultivated for 6 h at 30 °C and 250 rpm. Protein production was induced after 2 h of incubation with 200 μM IPTG (Invitrogen, Thermo Fisher Scientific). Normalized amounts of cells corresponding to 625 μl *A*_600_^−1^ were harvested at the end of the cultivation by centrifugation at 13,000*g* for 3 min at 4 °C. Cell pellets were resuspended in 240 μl Buffer Z (60 mM Na_2_HPO_4_, 40 mM NaH_2_PO_4_, 10 mM KCl, 1 mM MgSO_4_, and 50 mM β-mercaptoethanol, pH 7) and 5 μl lysozyme-DNase (8 mg mL^−1^ lysozyme and 1.25 mg mL^-1^ DNase) (Thermo Fisher Scientific), incubated for 10 min at 37 °C and centrifuged at 13,000*g* for 2 min. In total, 200 μl supernatant were mixed with 600 μl Buffer Z and reactions were started by addition of 200 μl ortho-nitrophenyl beta-D-galactopyranoside (Thermo Fisher Scientific) (4 mg mL^−1^, 19.5 mM NaH_2_PO_4_ and 30.5 mM Na_2_HPO_4_, pH 7) and incubation at 28 °C. When solutions turned yellow, incubation time was noted, and reactions were stopped by addition of 0.5 ml 1 M Na_2_CO_3_. The *A*_420_ was determined against a blank containing 800 μl Buffer Z subjected to the same reaction. β-galactosidase activity was calculated according to Eq. 1.

(1) $${Activity}_{\beta -Galactosidase}\left(MU\right)=\frac{1500\times {OD}_{420}}{0.5\times {Incubation\;time}_{\left(\min\right)}}$$

### Electrophoretic mobility shift assay

Double-stranded DNA fragments covering the putative promoters and their flanking regions were obtained by PCR using the appropriate primer pairs (Table S4) and plasmids pGK-P*phaZ3*-lacZ, pGK-P*phaZ5*-lacZ, and pMG7 as template. The fragments were intrinsically labeled with [α-^32^P]-dATP during the PCR. The DNA fragments were separated from unincorporated [α-^32^P]-dATP by passage through a Sephadex column (Sigma-Aldrich). Since intrinsic labeling of the P*phaP1* fragment was unsuccessful, oligo MG0235 was 5′ end-labeled with [γ-^32^P]-ATP using T4 polynucleotide kinase (New England Biolabs) according to the manufacturer’s instructions. Following purification from 15% (w/v) denaturing polyacrylamide gels, the oligonucleotide was annealed with the complementary unlabeled oligo MG0236 by incubation at 95 °C for 5 min and subsequent slow cooling to 30 °C. To ensure complete hybridization of the labeled oligos, the unlabeled oligo was added in a 2-fold excess.

For EMSAs, 13 μl master mix containing the labeled DNA (for 10 reactions: 50,000 cpm DNA, 20 μl 10× TBE, 110 μl glycerin-bromophenol blue and 0.8 μl 10 mg mL^−1^ herring sperm DNA (Invitrogen and Thermo Fisher Scientific)) were incubated with 17 μl protein dilutions for 15 min at 30 °C and loaded into a 6% (37.5:1) native polyacrylamide gel and run at 230 V and 25 mA at 4 °C. Dried gels were analyzed by PhosphorImaging in a Biostep PhosphorImager using AIDA Image Analyzer 5.0 software (Raytest).

## Data availability

The raw data supporting the conclusion of this article will be made available by the authors, without undue reservation. The mass spectrometry proteomics data have been deposited to the ProteomeXchange Consortium *via* the PRIDE partner repository (40) with the dataset identifier PXD050193 and 10.6019/PXD050193.

## Supporting information

This article contains supporting information (7, 39, 41).

## Conflict of interest

The authors declare that they have no conflicts of interest with the contents of this article.
