# Supplementary material for: Elucidating regulation of polyhydroxyalkanoate metabolism in Ralstonia eutropha: Identification of transcriptional regulators from phasin and depolymerase genes
Source: J Biol Chem. 2024 Jul 4;300(8):107523. doi: 10.1016/j.jbc.2024.107523 (PMC11332829; doi:10.1016/j.jbc.2024.107523)
Supplement: Supplementary material [file mmc1.docx]

*Supporting Information*

Elucidating regulation of polyhydroxyalkanoate metabolism in *R. eutropha*: Identification of transcriptional regulators from phasin and depolymerase genes

Lara Santolin^1^, Rosalie Sandra Josianne Eichenroth^1^, Paul Cornehl^1^, Henrike Wortmann^1^, Christian Forbrig^2^, Anne Schulze^2^, Inam Ul Haq^3^, Sabine Brantl^3^, Juri Rappsilber^2^, Sebastian Lothar Riedel^1,4^, Peter Neubauer^1^, Matthias Gimpel^1,*^

^1^ Technische Universität Berlin, Chair of Bioprocess Engineering, Berlin, Germany

^2^ Technische Universität Berlin, Chair of Bioanalytics, Berlin, Germany

^3^ Matthias-Schleiden-Institut für Genetik, Bioinformatik und Molekulare Botanik, AG Bakteriengenetik, Friedrich-Schiller-Universität Jena, Jena, Germany

^4^ Berliner Hochschule für Technik, Environmental and Bioprocess Engineering Laboratory, Berlin, Germany

***Table S1| Bacterial strains used in this study.***

| **Strain** | **Genotype** | **Reference** |
| --- | --- | --- |
| *Escherichia coli* DH5α | F^-^ *endA1 glnV44 thi-1 recA1 relA1 gyrA96 deoR*  *nupG purB20 φ80dlacZΔM15 Δ(lacZYA-argF)U169 hsdR17(r_K_^–^m_K_^+^) λ-* | (Hanahan, 1985) |
| *E. coli* BL21 Gold | F^-^*ompT hsdS(r_B–_ m_B–)_ dcm^+^ Tet^R^ gal endA The* | Agilent, Waldbronn |
| *Ralstonia eutropha* Re2058/pCB113 | Re2058:H16 Δ*phaC1* Δ*proC*  pCB113: pBBR1MCS-2 with *phaC2_Ra_*  *phaA*_H16_ *phaJ1*_Pa_ *proC*_H16_ Km^R^ | (Budde et al., 2011) |

***Table S2 |Identificators of phasins- and depolymerases gene sequences studied.*** *NCBI data bank Accesion number, localization in the genome, locus tag and protein id is given for every gene.*

| **Gene** | **Accesion** | **Localization** | **Locus tag** | **Protein Id** |
| --- | --- | --- | --- | --- |
| phaP1 | [AM260479](https://www.ncbi.nlm.nih.gov/nuccore/AM260479) | Chromosome 1 | H16_A1381 | [CAJ92517.1](https://www.ncbi.nlm.nih.gov/protein/113526172) |
| phaP2 | [AY305378](https://www.ncbi.nlm.nih.gov/nuccore/AY305378) | Megaplasmid pHG1 | PHG202 | [AAP85954.1](https://www.ncbi.nlm.nih.gov/protein/32527204) |
| phaP3 | [AM260479](https://www.ncbi.nlm.nih.gov/nuccore/AM260479) | Chromosome 1 | H16_A2172 | [CAJ93269.1](https://www.ncbi.nlm.nih.gov/protein/113526924) |
| phaP4 | [AM260480](https://www.ncbi.nlm.nih.gov/nuccore/AM260480) | Chromosome 2 | H16_B2021 | [CAJ96803.1](https://www.ncbi.nlm.nih.gov/protein/113530456) |
| phaP5 | [AM260480](https://www.ncbi.nlm.nih.gov/nuccore/AM260480) | Chromosome 2 | H16_B1934 | [CAJ96716.1](https://www.ncbi.nlm.nih.gov/protein/113530369) |
| phaP6 | AM260480 | Chromosome 2 | H16_B1988 | [CAJ96770.1](https://www.ncbi.nlm.nih.gov/protein/113530423) |
| phaP7 | AM260480 | Chromosome 2 | H16_B2326 | [CAJ97108.1](https://www.ncbi.nlm.nih.gov/protein/113530761) |
| phaZ1 | [AM260479](https://www.ncbi.nlm.nih.gov/nuccore/AM260479) | Chromosome 1 | H16_A1150 | [CAJ92291.1](https://www.ncbi.nlm.nih.gov/protein/113525946) |
| phaZ2 | [AM260479](https://www.ncbi.nlm.nih.gov/nuccore/AM260479) | Chromosome 1 | H16_A2862 | [CAJ93939.1](https://www.ncbi.nlm.nih.gov/protein/113527594) |
| phaZ3 | AM260480 | Chromosome 2 | H16_B0339 | [CAJ95139.1](https://www.ncbi.nlm.nih.gov/protein/113528792) |
| phaZ4 | [AY305378](https://www.ncbi.nlm.nih.gov/nuccore/AY305378) | Megaplasmid pHG1 | PHG178 | [AAP85930.1](https://www.ncbi.nlm.nih.gov/protein/32527180) |
| phaZ5 | AM260480 | Chromosome 2 | H16_B1014 | [CAJ95805.1](https://www.ncbi.nlm.nih.gov/protein/113529458) |
| phaZ6 | AM260480 | Chromosome 2 | H16_B2073 | [CAJ96855.1](https://www.ncbi.nlm.nih.gov/protein/113530508) |
| phaZ7 | AM260480 | Chromosome 2 | H16_B2401 | [CAJ97183.1](https://www.ncbi.nlm.nih.gov/protein/113530836) |
| phaY1 | [AM260479](https://www.ncbi.nlm.nih.gov/nuccore/AM260479) | Chromosome 1 | H16_A2251 | [CAJ93348.1](https://www.ncbi.nlm.nih.gov/protein/113527003) |
| phaY2 | [AM260479](https://www.ncbi.nlm.nih.gov/nuccore/AM260479) | Chromosome 1 | H16_A1335 | [CAJ92475.1](https://www.ncbi.nlm.nih.gov/protein/113526130) |

***Table S3 | Plasmids used in this study.***

| **Plasmid** | **Description** | **Reference** |
| --- | --- | --- |
| pMG7 | Vector for integration into *B. subtilis amyE*; P*_cggR_*, gapA 5′UTR, strep-tag, ColE1 ori, Amp^R^, Cm^R^ | Gimpel & Brantl, 2012 |
| pGW5 | *E. coli* expression vector, P*_Lac_*, LacO3, LacO1, strep-tag, *Bacillus subtilis* BsrF terminator, ColE1 ori, *parB*, Amp^R^ | Gimpel, unpublished |
| pGW5-B0227 | As pGW5, with *R. eutropha H16_B0227* gene | This work |
| pGW5-B1672 | As pGW5, with *R. eutropha H16_B1672* gene | This work |
| pGW5-B2256 | As pGW5, with *R. eutropha H16_B2256* gene | This work |
| pGW5-PhaA | As pGW5, with *R. eutropha phaA* gene | This work |
| pGW5-PhaP1 | As pGW5, with *R. eutropha phaP1* gene | This work |
| pGW5-PpiB | As pGW5, with *R. eutropha ppiB* gene | This work |
| pGK-LacZ | *E. coli/ B. subtilis* shuttle vector, pWV01 ori, promoterless *lacZ* gene, Spec^R^ | Gimpel, unpublished |
| pGK-PphaZ3-lacZ | As pGK-LacZ, with *R. eutropha* P*_phaZ3_* | This work |
| pGK-PphaZ5-lacZ | As pGK-LacZ, with *R. eutropha* P*_phaZ5_* | This work |
| pGK-PphaP1-lacZ | As pGK-LacZ, with *R. eutropha* P*_phaP1_* | This work |
| pGK-PcggR-LacZ. | As pGK-LacZ, with *B. subtilis* P*_cggR_* | This work |

***Table S4: Oligonucleotides used in this study.***

| Name | Sequence (starting with 5‘ end) | Purpose |
| --- | --- | --- |
| MG0264 | 5’ ATC GGA TCC ATG GCG ACG TAT CAG GAA ATC | C, pGW5-B0227 |
| MG0265 | 5’ ATC AAG CTT AGC GGA TCA GGA AGT CTT CAA | C, pGW5-B0227 |
| MG0290 | 5’ ATC GGA TCC ATG AAC AGT ACA GCC GTG CCA | C, pGW5-B1672 |
| MG0291 | 5’ ATC AAG CTT AGA CCG GCT TGT GGC CCT CTC | C, pGW5-B1672 |
| MG0288 | 5’ ATC GGA TCC ATG GCG ACA TAC AAG CAA CTC | C, pGW5-B2256 |
| MG0289 | 5’ ATC AAG CTT ACT GCT CGA TCA GGA AAC G | C, pGW5-B2256 |
| MG0258 | 5’ ATC GGA TCC ATG ACT GAC GTT GTC ATC GTA | C, pGW5-PhaA |
| MG0259 | 5’ ATC AAG CTT ATT TGC GCT CGA CTG CCA G | C, pGW5-PhaA |
| MG0292 | 5’ ATC GGA TCC ATG ATC CTC ACC CCG GAA CAA | C, pGW5-PhaP1 |
| MG0293 | 5’ ATC AAG CTT AGG CAG CCG TCG TCT TCT T | C, pGW5-PhaP1 |
| MG0298 | 5’ ATC GGA TCC ATG TCC AAG GTC CAG CTC CAC | C, pGW5-PpiB |
| MG0299 | 5’ ATC AAG CTT ACT CGA CGA TGA CGG CCT T | C, pGW5-PpiB |
| MG0278 | 5’ ACT GAA TTC GAC CGG TAC GGG GTC GCA AG | C, pGK-PphaZ3-lacZ |
| MG0279 | 5’ ACT GGA TCC GCC AGG ATT TCA CTA TAT GTC | C, pGK-PphaZ3-lacZ |
| MG0310 | 5’ ATC GAA TTC TGG TCG GCA TAG AAA GCG AGC | C, pGK-PphaP1-lacZ S |
| MG0341 | 5’ TAT TTT AGG GGC GCC AGA TCC AAT GTC AAG …..TTG ATT TTG TGC AAT GCA | C, pGK-PphaP1-lacZ |
| MG0342 | 5’ ATC CTG CAG GGA TCC GCG CCG CAA CAA TTC …..CTA TTT TAG GGG CGC CAG ATC CA | C, pGK-PphaP1-lacZ |
| MG0343 | 5’ ACT GGA TCC ATT TCT GCT TTC TTT AAT TAT | C, pGK-PcggR-lacZ |
| MG0346 | 5’ ATC GAA TTC TTT GGA TAA AAT GAA AAC GCT | C, pGK-PccgR-lacZ |
| MG0235 | 5’ TGG CGT CAC AGC CGC TCC CGT GTA TCG CCA …….GCA ACG TTG TTT GTG CAT TGC ACA AAA TCC …….ACT TGA CAT TGG ATC TGG CGC CCC TAA AAT …….AGG AAT TGT T [Btn] | P & E, PphaP1 |
| MG0236 | 5’ AAC AAT TCC TAT TTT AGG GGC GCC AGA TCC …….AAT GTC AAG TGG ATT TTG TGC AAT GCA CAA ………ACA ACG TTG CTG GCG ATA CAC GGG AGC GGC ………TGT GAC GCC A | P & E, PphaP1 |
| MG0239 | 5’ GAC CGG TAC GGG GTC GCA AGC AAG CGG GCC …….CCG CCA GCG GCG CGG CGC TTG ATG TTT GGC …….GTT GCA GCA AGG CCT GTC CGA CAT ATA GTG …….AAA TCC TGG C [Btn] | P & E, PphaZ3 |
| MG0240 | 5’ GCC AGG ATT TCA CTA TAT GTC GGA CAG GCC …….TTG CTG CAA CGC CAA ACA TCA AGC GCC GCG …….CCG CTG GCG GGG CCC GCT TGC TTG CGA CCC …….CGT ACC GGT C | P & E, PphaZ3 |
| MG0241 | 5’ ACC TAC GCA CCC GCG GCA GAA GCC TGT CGC …….CCG CCC GCC CAC CTT GCA CGA AGC CGG CCA …….TGC GGC TGT CGG GCA CGC CAA CTG CGC TGA …….AGC CCG CAT T [Btn] | P & E, PphaZ5 |
| MG0242 | 5’ AAT GCG GGC TTC AGC GCA GTT GGC GTG CCC …….GAC AGC CGC ATG GCC GGC TTC GTG CAA GGT …….GGG CGG GCG GGC GAC AGG CTT CTG CCG CGG …….GTG CGT AGG T | P & E, PphaZ5 |
| MG0272 | 5’ TGG CGT CAC AGC CGC TCC CGT | Shift P1 |
| MG0275 | 5’ GCC AGG ATT TCA CTA TAT GTC | Shift Z3 |
| MG0311 | 5’ ATC GAA TTC GGA CCC GCG TCC GAG GTA T | Shift Z3 |
| MG0370 | 5’ ATC GAA TTC GAC CGG AGC CTC CCG CAG CCA | C, pGK-PphaZ5-lacZ; Shift Z5 |
| MG0372 | 5’ ATC GGA TCC GGG TGC TGC GCA ATG CG | C, pGK-PphaZ5-lacZ Shift Z5 |
| SB3847 | 5’ ATT TCT GCT TTC TTT AAT | Shift cggr |
| SB3850 | 5’ TTT GGA TAA AAT GAA AAC GCT TTA | Shift cggr |
| M13-24R | CGG ATA ACA ATT TCA CAC AGG | S |

S, sequencing; C, construction of plasmid followed by plasmid designation


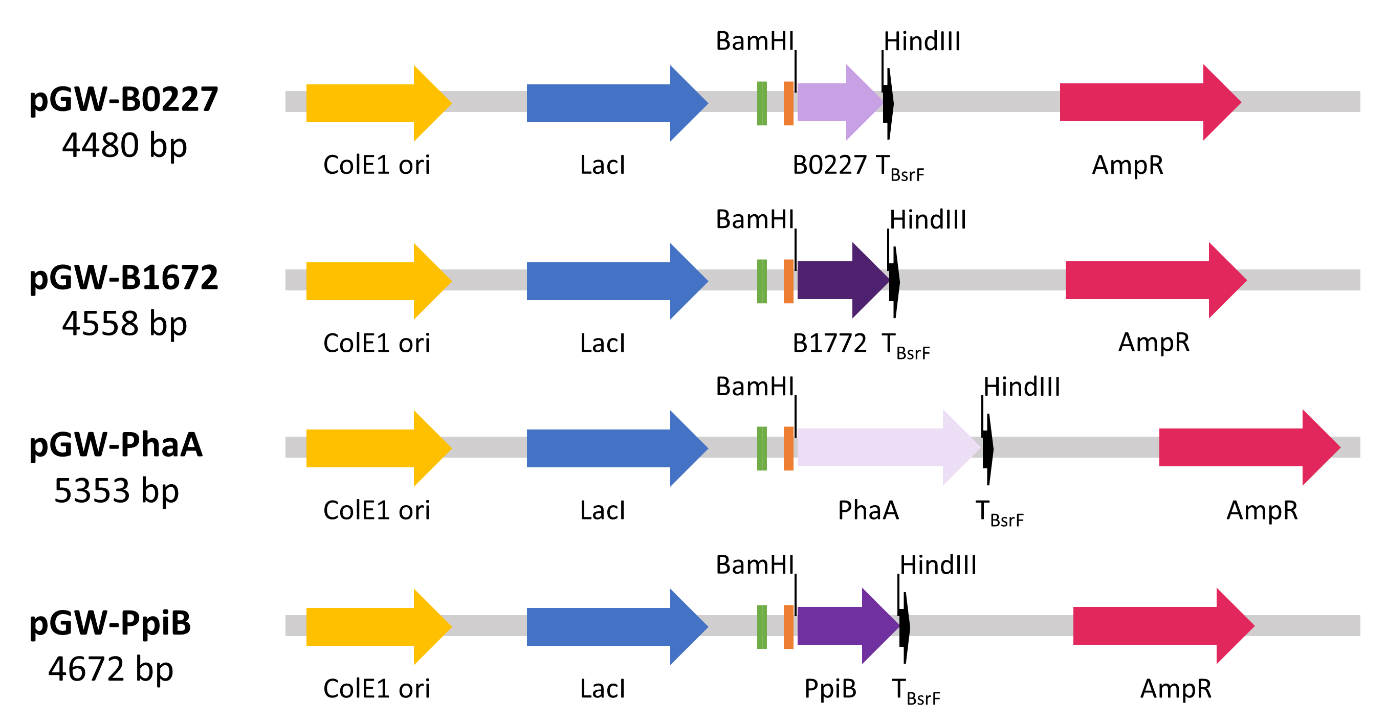


***Figure S1| Plasmid maps of pGW5 derivates.*** *For better clarity plasmids are depicted in a linearized form. Genes H16_ B0227, H16_B1672, PhaA and PppiB were inserted between the -35/-10 region (light green) and the TbsrF terminator using the restriction enzymes BamHI and HindIII. An upstream STREP-Tag (orange) enables purification through affinity chromatography. Additionally, pGW5 derivatives possess an ampicillin resistance gene for the selection of transformed cells. The expression of the inserts is controlled by the Lac repressor LacI and can be specifically induced with IPTG. the Copy number of pGW5 derivatives is regulated by the ColE1 replication origin.*


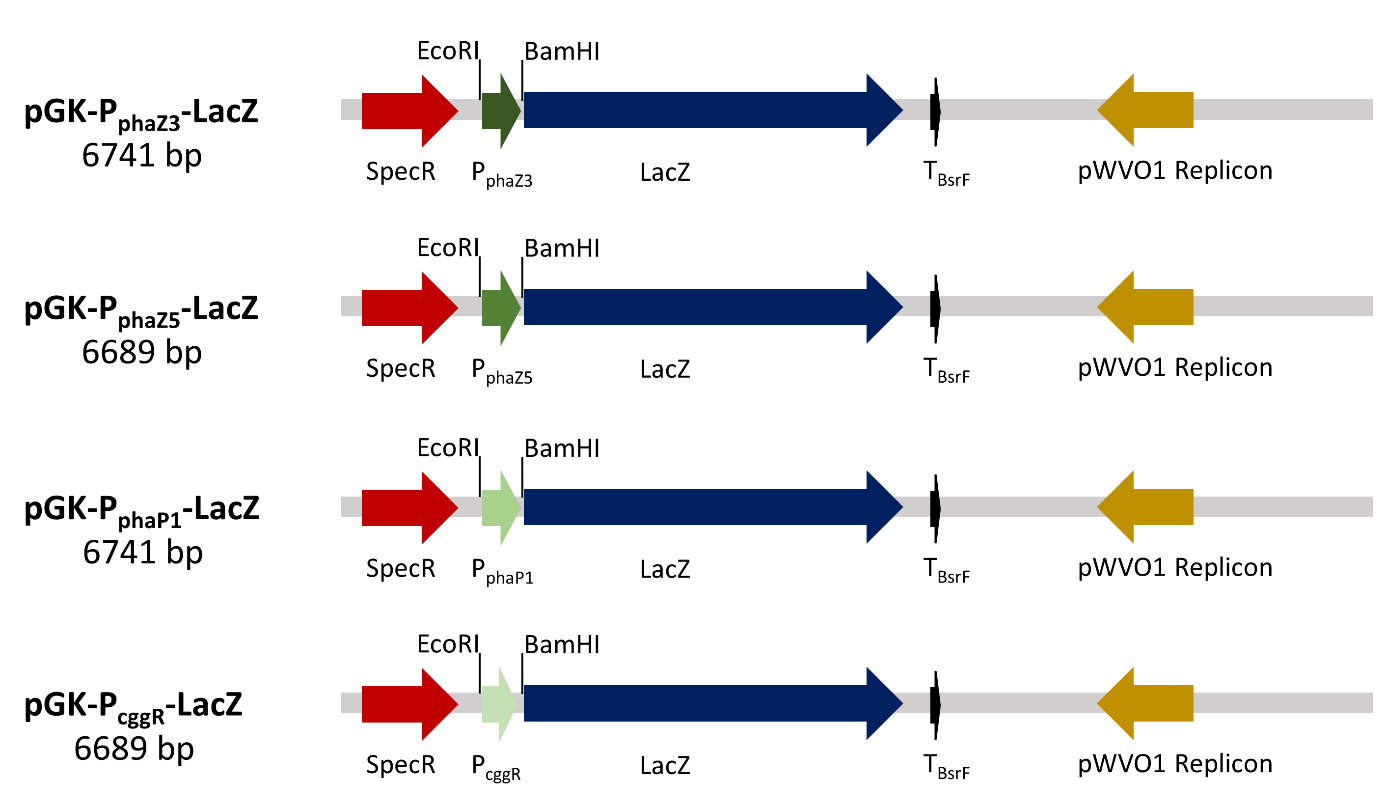


***Figure S2| Plasmid maps of pGK-LacZ derivates****. For better clarity plasmids are depicted in a linearized form. The R. eutropha promoters PphaZ3, PphaZ5 and PphaP1 and the B. subtilis control promoter PpcggR were inserted upstream of the reporter gene LacZ using the restriction enzymes EcoRI and BamHI. Selection of cells transformed with pGK derivatives was achieved through a Spectinomycin resistance gene. Replication of pGK derivatives is controlled by the pWVO1 replicon.*

*
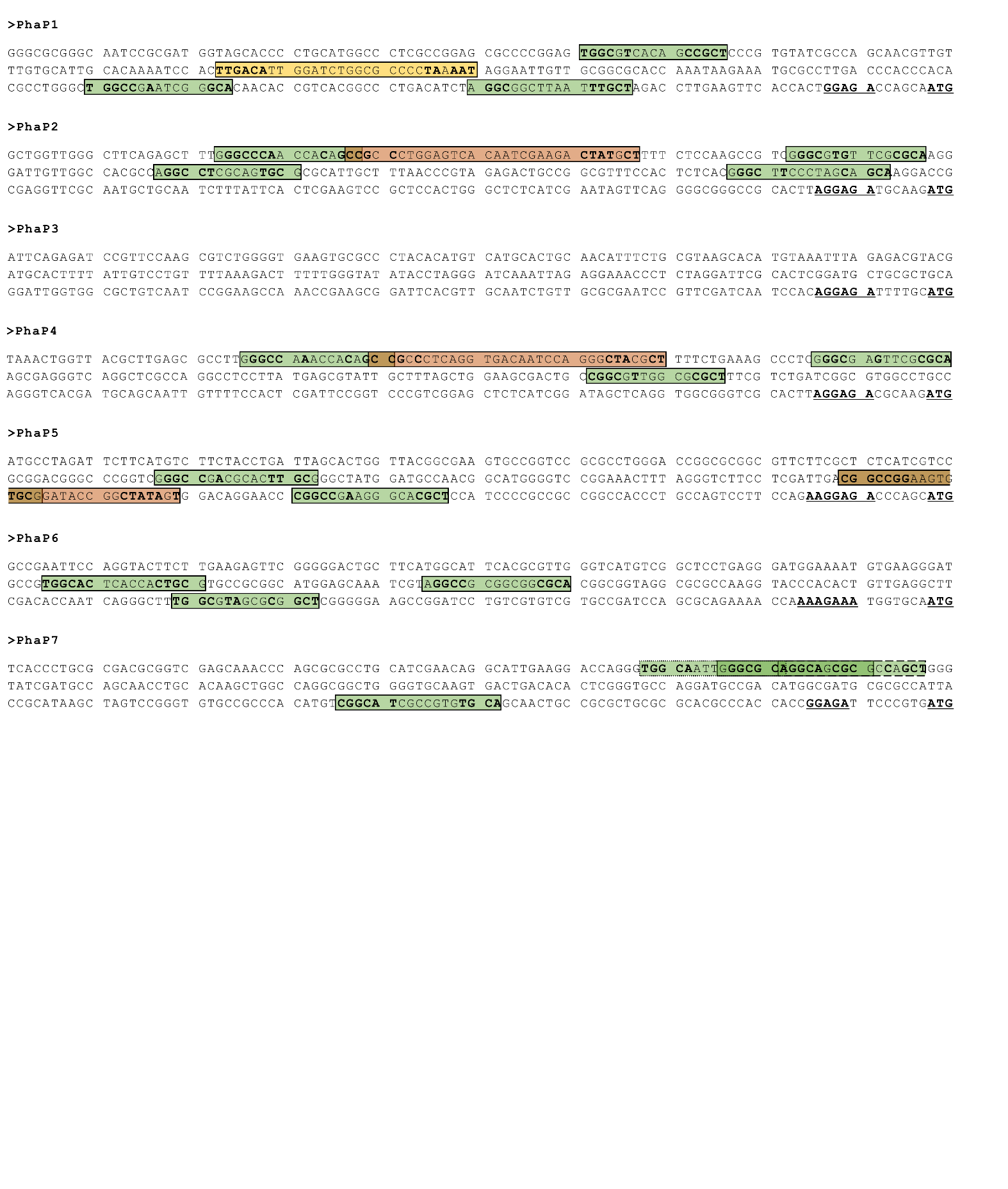
*

***Figure S3 |Putative promoters identified for phasin genes of R. eutropha H16.*** *σ^70^, σ^N^, σ^S^ - consensus regions are highlighted in yellow, green and red, respectively. The identical nucleotides in each sequence to the consensus sequences are highlighted in black.*

*
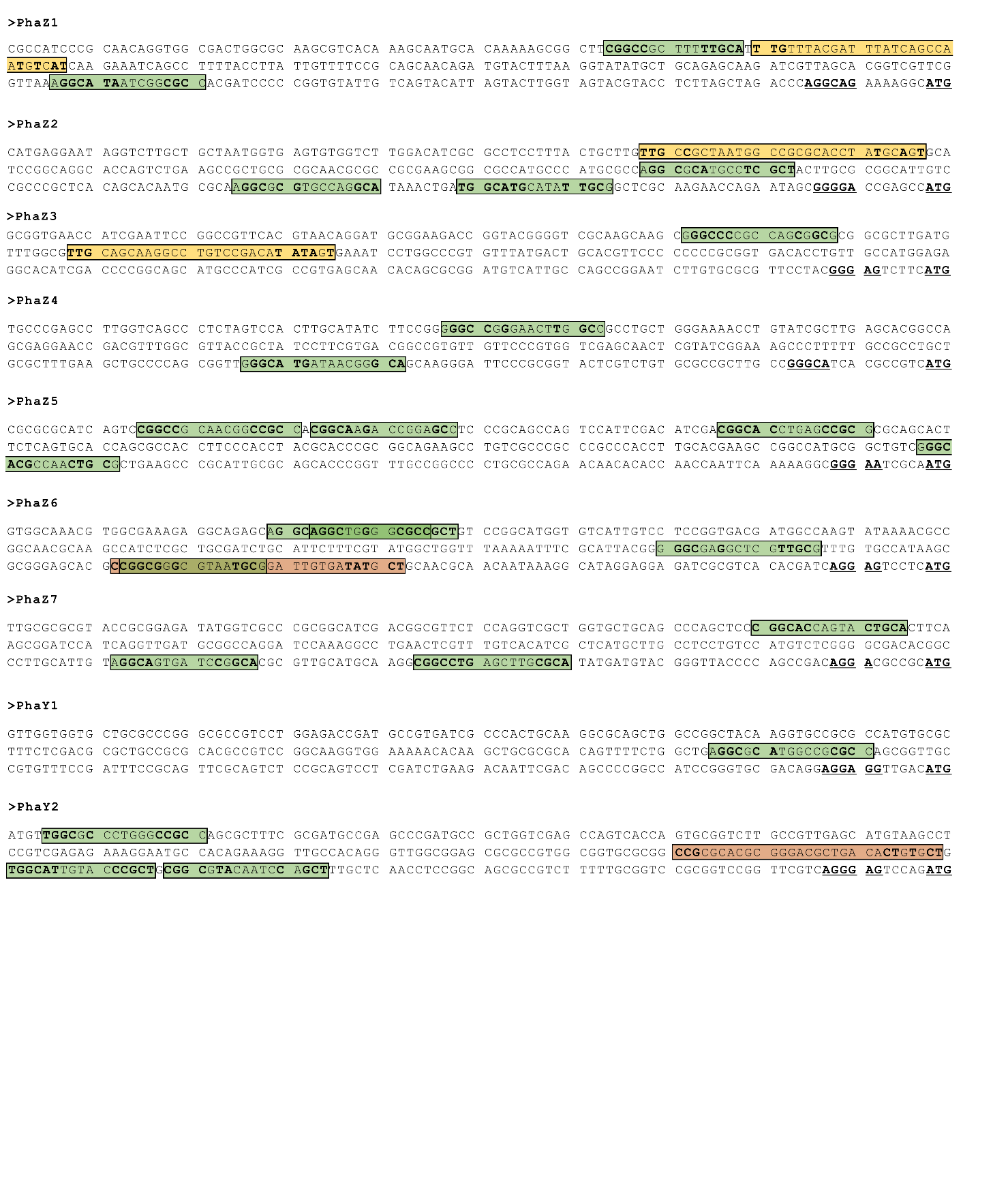
* ***Figure S4 |Putative promoters identified for depolymerase genes of R. eutropha H16****. σ^70^, σ^N^, σ^S^ - consensus regions are highlighted in yellow, green and red, respectively. Identical nucleotides in each sequence to the consensus sequences are highlighted in black.*

***
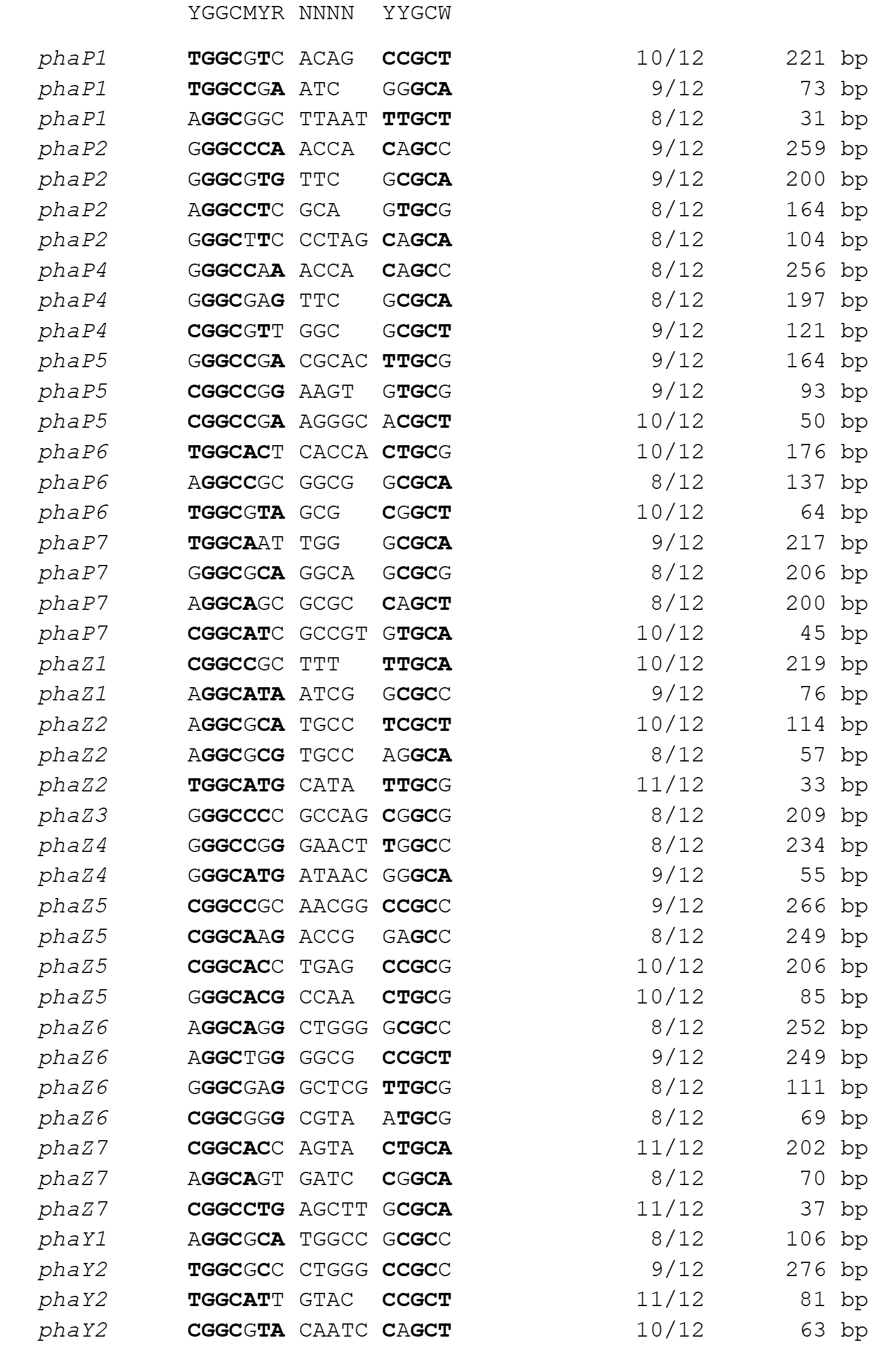
***

***Figure S5 |Alignment of* σ^N^*-like consensus promoter regions of phasin and depolymerase genes of R. eutropha H16.*** *First row, consensus* σ^N^*- promoter region in IUPAC nomenclature. First column, gene; second column, putative promoter sequence. The identical nucleotides in each sequence to the consensus sequences are highlighted in black. Third column, consensus score. Fourth column, distance to the start codon.*


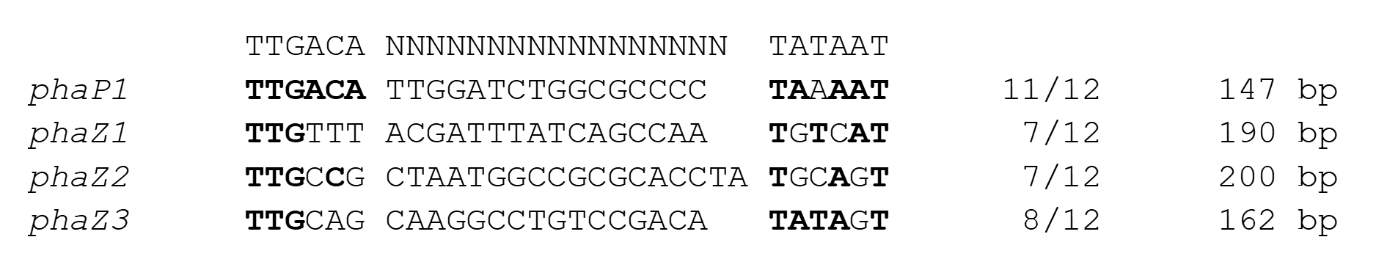


***Figure S6 |Alignment of* σ^70^ *consensus promoter region with sequences upstream phasin and depolymerase genes of R. eutropha H16****. First row, consensus σ^70^- region in IUPAC nomenclature. First column, gene; second column, putative promoter sequence. The identical nucleotides in each sequence to the consensus sequences are highlighted in black. Third column, consensus score. Fourth column, distance to the start codon.*


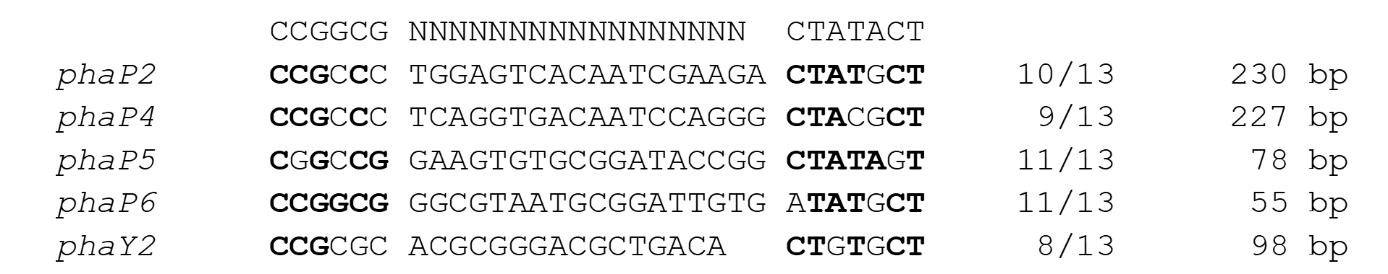


***Figure S7 |Alignment of* σ^S^ *consensus promoter region with sequences upstream phasin and depolymerase genes of R. eutropha H16****. First row, consensus* σ^S^*- promoter region in IUPAC nomenclature. First column, gene; second column, putative promoter sequence. The identical nucleotides in each sequence to the consensus sequences are highlighted in black. Third column, consensus score. Fourth column, distance to the start codon.*


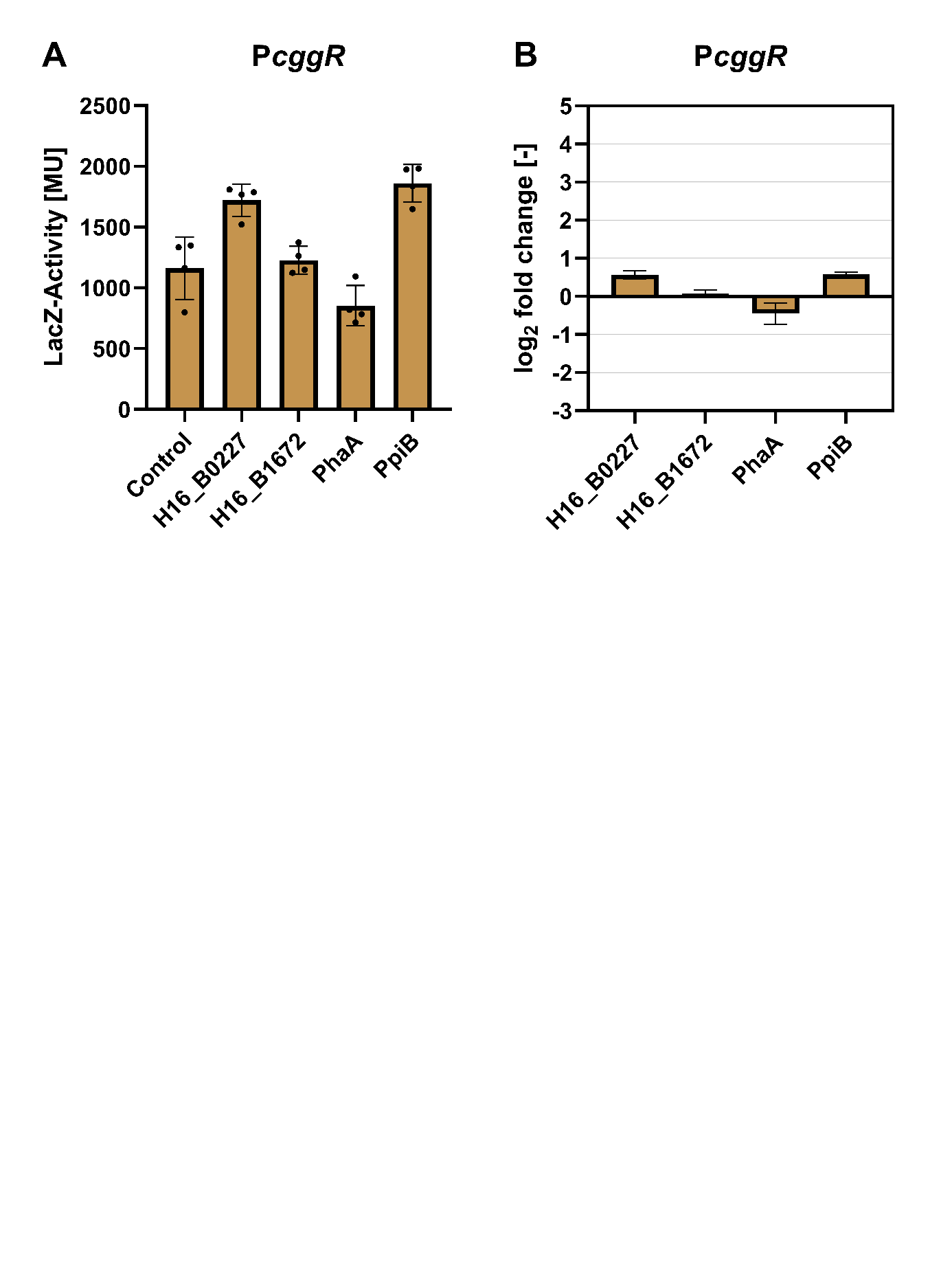


***Figure S8 | Control β-galactosidase reporter-gene assay to test unspecific effects of proteins H16_B0227, H16_B1672, PhaA or PpiB on promoter PcggR****. A: The measured β-galactosidase activity is displayed for each protein as well as the control with an empty plasmid for the putative TF. Scatter plots (black dots) are shown to report the reproducibility of 4 independent biological replicates within our data sets. B: The measured β-galactosidase activity is presented as a logarithmic fold change against a control with an empty plasmid for the putative TF. Error bars represent the standard deviation of 4 biological replicates.*


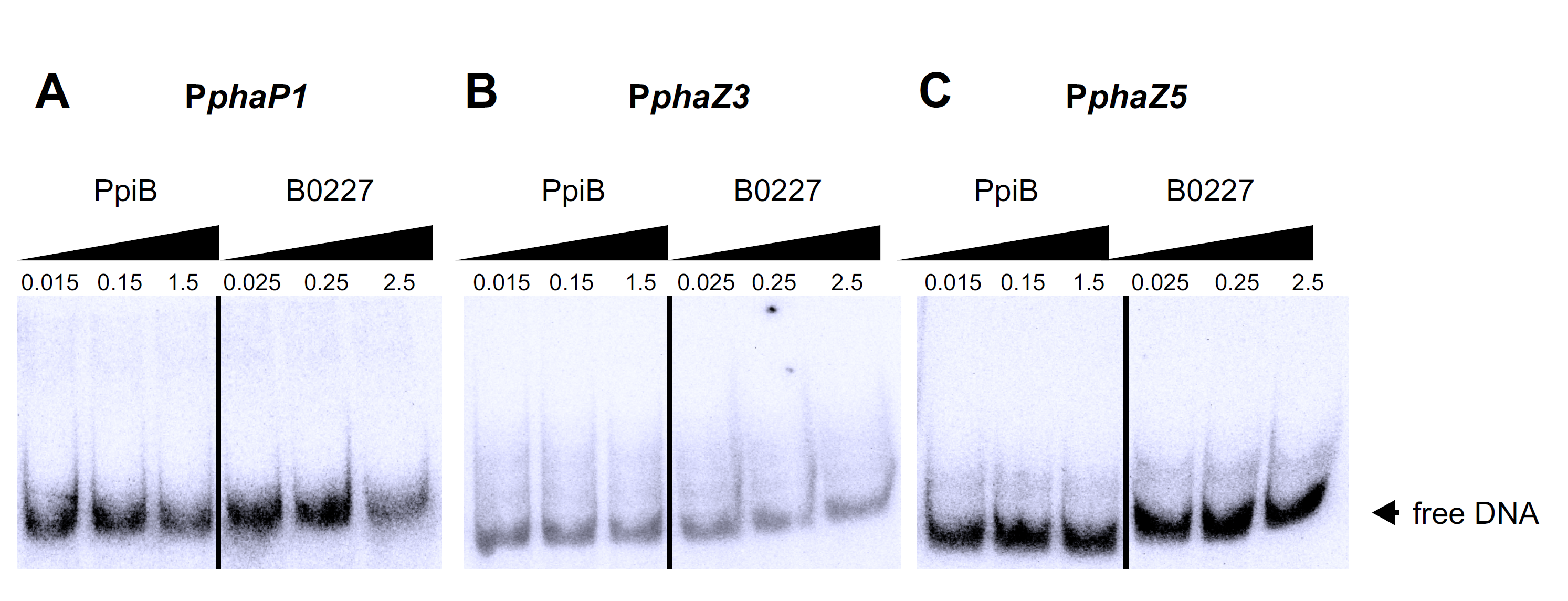


***Figure S9 | EMSA with putative TF proteins PpiB and H16_B0227 with phasin promoter PphaP1 (A) and depolymerase promoters PphaZ3 (B) and PphaZ5 (C). Black vertical lines indicate splice positions.***

**

***Figure S10 | EMSA with putative TF protein H16_B1672 and depolymerase promoter PphaZ3.***

******

***Figure S11 | Control EMSA to test unspecific binding of H16_B1672, PpiB and H16_B0227 to the heterologous B. subtilis promoter PccgR. The black vertical line indicates a splice position.***
